# Increased EphA4-ephexin1 signaling in the medial prefrontal cortex plays a role in depression-like phenotype

**DOI:** 10.1038/s41598-017-07325-2

**Published:** 2017-08-02

**Authors:** Ji-chun Zhang, Wei Yao, Youge Qu, Mayumi Nakamura, Chao Dong, Chun Yang, Qian Ren, Min Ma, Mei Han, Yukihiko Shirayama, Akiko Hayashi-Takagi, Kenji Hashimoto

**Affiliations:** 1 Division of Clinical Neuroscience, Chiba University Center for Forensic Mental Health, Chiba, 260-8670 Japan; 2 Laboratory of Medical Neuroscience, Institute for Molecular and Cellular Regulation, Gunma University, Gunma, 371-8511 Japan; 3 Department of Psychiatry, Teikyo University Chiba Medical Center, Ichihara Chiba, 299-0111 Japan; 4 PRESTO, Japan Science and Technology Agency, 4-1-8 Honcho, Kawaguchi Saitama, 332-0012 Japan

**Keywords:** Depression, Depression

## Abstract

Accumulating evidence suggests a role of the ephrin receptor EphA4 and the downstream protein ephexin1 in synaptic plasticity, which is implicated in depression. We examined whether EphA4–ephexin1 signaling plays a role in the pathophysiology of depression, and the antidepressant-like effect of EphA4 inhibitor rhynchophylline. We found increased ratios of p-EphA4/EphA4 and p-ephexin1/ephexin1 in the prefrontal cortex (PFC) and hippocampus but not in the nucleus accumbens (NAc), of susceptible mice after social defeat stress. Furthermore, the p-EphA4/EphA4 ratio was higher in the parietal cortex of depressed patients compared with controls. Systemic administration of rhynchophylline, produced a rapid antidepressant-like effect in a social defeat stress model by inhibiting EphA4–ephexin1 signaling and activating brain-derived neurotrophic factor-TrkB signaling in the PFC and hippocampus. Pretreatment with rhynchophylline before each social defeat stress could prevent the onset of the depression-like phenotype after repeated social defeat stress. Overexpression of EphA4 in the medial PFC owing to infection with an EphA4 adeno-associated virus caused the depression-like phenotype 3 weeks later and rhynchophylline had a rapid antidepressant-like effect in these mice. These findings suggest that increased EphA4–ephexin1 signaling in the PFC plays a role in the pathophysiology of depression.

## Introduction

Depression is the most prevalent psychiatric disorder and one of the most severe and debilitating disorders. The World Health Organization estimates that more than 350 million individuals of all ages suffer from depression^1^. The antidepressants currently available are generally effective in the treatment of depression, but it may take weeks before patients feel the full antidepressant effects^2–4^. Furthermore, approximately one-third of the depressed patients fail to respond to pharmacotherapy, there is a high rate of relapse, and patients with depression also have a high risk of attempting suicide. Therefore, the development of novel antidepressants targeting the neuropathological mechanisms underlying the disorder is required^5, 6^.

The erythropoietin-producing hepatocellular (Eph) receptor family has 14 members in mammals and is divided into two classes or receptors, EphA (A1–A8 and A10) and EphB (B1–B4 and B6), based on their sequence homology and binding affinities with ephrinA (A1–A5) and ephrinB (B1–B3) ligands^7–9^, respectively. EphA4 is the EphA receptor family member with the highest expression level in the adult hippocampus where it plays a role in synaptic plasticity^10–12^. It is known that EphA4 acts as a negative regulator of *N*-methyl-D-aspartate (NMDA) receptor neurotransmission^13, 14^, which is implicated in the pathophysiology of depression^15–17^. Emerging evidence also suggests that Eph receptors and their ligands, ephrins, play roles in aberrant synaptic functions associated with cognitive impairments in patients with neurodegenerative disorders^18^. In addition, EphA4 can enhance the activation of ephexin1, which is a guanine-nucleotide exchange factor that regulates the activation of the small Ras-homologous (Rho) GTPase RhoA^19–21^. EphA4-dependent spine retraction through the activation of ephexin1 is implicated in the regulation of the post-synaptic structure as well as pre-synaptic vesicle release at various types of synapses^19–21^. It is well known that changes in the dendritic length and spine density in the prefrontal cortex (PFC) and hippocampus contribute to the neurobiology of depression and that the action of antidepressants is mediated by blocking or reversing these changes^22–24^. Therefore, it is important to determine whether EphA4-ephexin1 signaling plays a role in the pathophysiology of depression.

In the present study, we examined the role of EphA4-ephexin1 signaling in the pathophysiology of depression. First, we tested whether the expression of EphA4 and ephexin1 as well as their phosphorylated forms was altered in the brain regions of mice with a depression-like phenotype and depressed patients. Second, we examined the effects of a novel EphA4 inhibitor, rhynchophylline^25–27^, and demonstrated its antidepressant-like effect in a social defeat stress model of depression. Third, we examined whether pretreatment with rhynchophylline before each stress could prevent the onset of the depression-like phenotype after repeated social defeat stress. Finally, we determined whether overexpression of EphA4-venus by adeno-associated virus (AAV) infection in the medial PFC caused the depression-like phenotype. Subsequently, we examined the antidepressant-like effect of rhynchophylline in mice after EphA4 AAV infection.

## Results

### Increased phosphorylation of EphA4 and ephexin1 in the susceptible rodents and the postmortem brains of depressed patients

**Figure 1:**
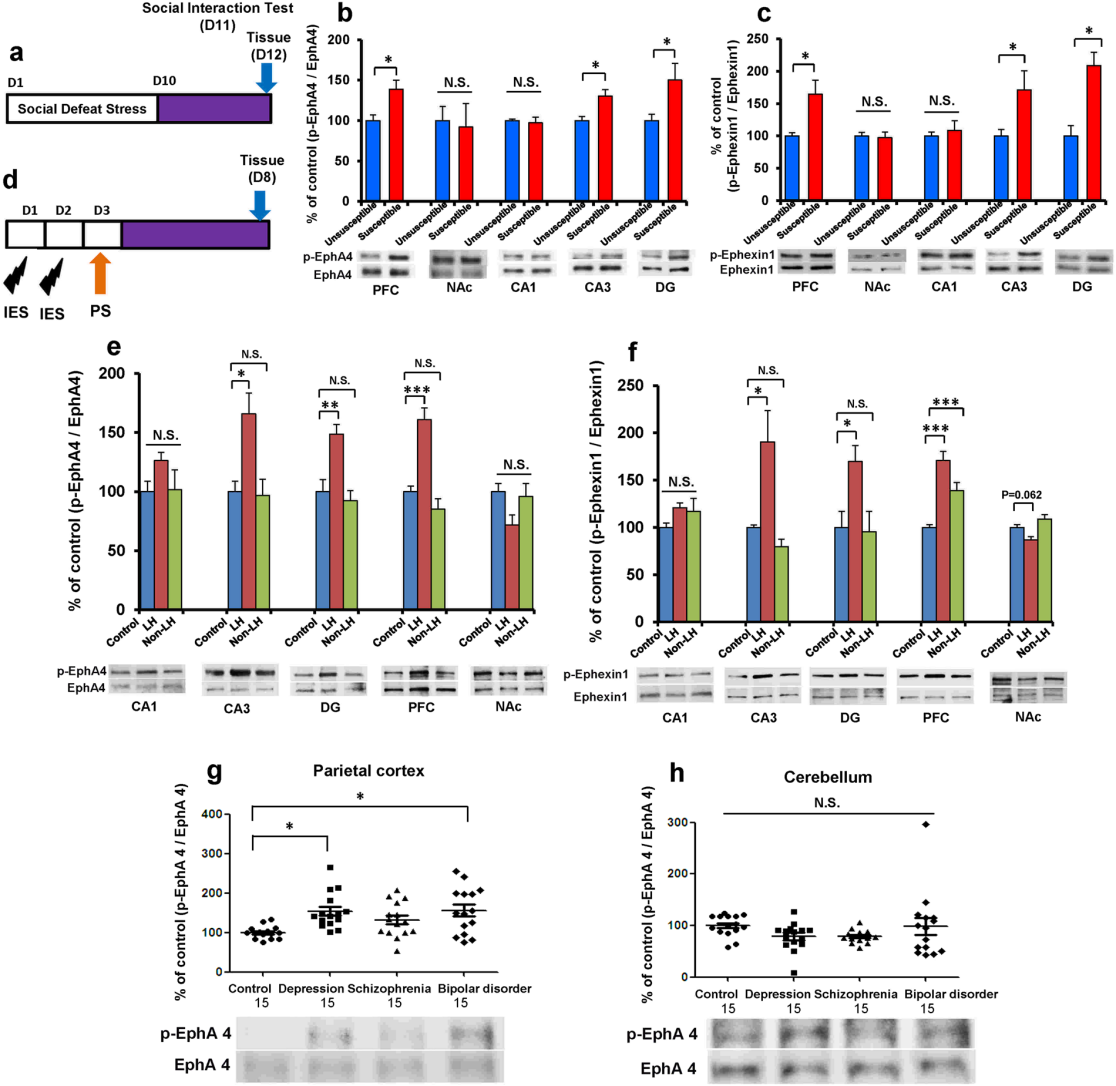
p-EphA4/EphA4 and p-ephexin1/ephexin1 ratios in the brain regions of mice with the depression-like phenotype and the postmortem brains of depressed patients. (**a**) Schedule of social defeat stress (10 days), social interaction test and brain sample collection. (**b** and **c**) The ratios of p-EphA4/EphA4 and p-ephexin1/ephexin1 in the mouse brain regions from social defeat stress (susceptible) mice and control (unsusceptible) mice. Data represent the mean ± S.E.M. (n = 5 or 6). *P < 0.05 compared with the control group. N.S.: not significant (Student’s *t*-test). (**d**) Schedule of learned helplessness (LH) and brain sample collection. (**e** and **f**) The ratios of p-EphA4/EphA4 and p-Ephexin1/Ephexin1 in the rat brain regions from control, LH, and non-LH rats (n = 5–6). Data represent the mean ± S.E.M. *P < 0.05, **P < 0.01 and ***P < 0.01 compared with the control group. N.S.: not significant. (**g** and **h**): The ratios of p-EphA4/EphA4 in the postmortem brain samples (parietal cortex and cerebellum) from control, depression, schizophrenia, and bipolar disorder subjects. Data represent the mean ± S.E.M. (n = 15). *P < 0.05 compared with control group, N.S.: not significant.

To clarify whether EphA4-ephexin1 signaling plays a role in the pathophysiology of depression, we performed Western blot for EphA4, ephexin1, and their phosphorylated forms (p-EphA4 and p-ephexin1) in susceptible mice after social defeat stress (Fig. 1a). Social defeat stress significantly increased the p-EphA4/EphA4 and p-ephexin1/ephexin1 ratios in the PFC (p-EphA4/EphA4 ratio: P = 0.018, p-ephexin1/ephexin1 ratio: P = 0.014), CA3 (p-EphA4/EphA4 ratio: P = 0.010, p-ephexin1/ephexin1 ratio: P = 0.045), dentate gyrus (DG) (p-EphA4/EphA4 ratio: P = 0.044, p-ephexin1/ephexin1 ratio: P = 0.002) of the hippocampus (Fig. 1b and c). By contrast, social defeat stress did not affect the ratio in the CA1 (p-EphA4/EphA4 ratio: P = 0.712, p-ephexin1/ephexin1 ratio: P = 0.606) and nucleus accumbens (NAc) (p-EphA4/EphA4 ratio: P = 0.819, p-ephexin1/ephexin1 ratio: P = 0.801) (Fig. 1b and c).

Furthermore, the p-EphA4/EphA4 and p-epexin1/ephexin1 ratios were significantly higher in the PFC, CA3, and DG from learned helplessness (LH: susceptible) rats compared with the control rats, but not in the non-LH (resilience) rats, (Fig. 1d–f). By contrast, LH stress did not change the ratio in the CA1 and NAc of rats (Fig. 1e and f). One-way ANOVA revealed statistical results for EphA4 (PFC: F_1,16_ = 23.882, P < 0.001; NAc: F_1,16_ = 2.548, P = 0.114, CA1: F_1,16_ = 1.503, P = 0.256; CA3: F_1,16_ = 7.583, P = 0.006; DG: F_1,16_ = 11.730, P = 0.001), and for ephexin1 (PFC: F_1,16_ = 20.591, P < 0.001; NAc: F_1,16_ = 8.215, P = 0.004, CA1: F_1,16_ = 1.406, P = 0.278; CA3: F_1,16_ = 7.994, P = 0.005; DG: F_1,16_ = 4.941, P = 0.024).

Using postmortem brain samples from the Neuropathology Consortium of the Stanley Medical Research Institute^28, 29^, we found that the p-EphA4/EphA4 ratio was significantly higher in the parietal cortex (Brodmann area 7: BA7) of subjects with depression and bipolar disorder, but not schizophrenia, than in that of controls (one-way ANOVA, F_1,59_ = 5.302, P = 0.003) (Fig. 1g). In contrast, there were no differences in the cerebellum among the four groups (one-way ANOVA, F_1,59_ = 1.534, P = 0.216) (Fig. 1h). These findings suggest that the increased p-EphA4/EphA4 ratio in the parietal cortex may be implicated in the pathogenesis of mood disorders such as depression and bipolar disorder.

### Rhynchophylline had antidepressant-like effects and prophylactic effects in a social defeat stress model

**Figure 2:**
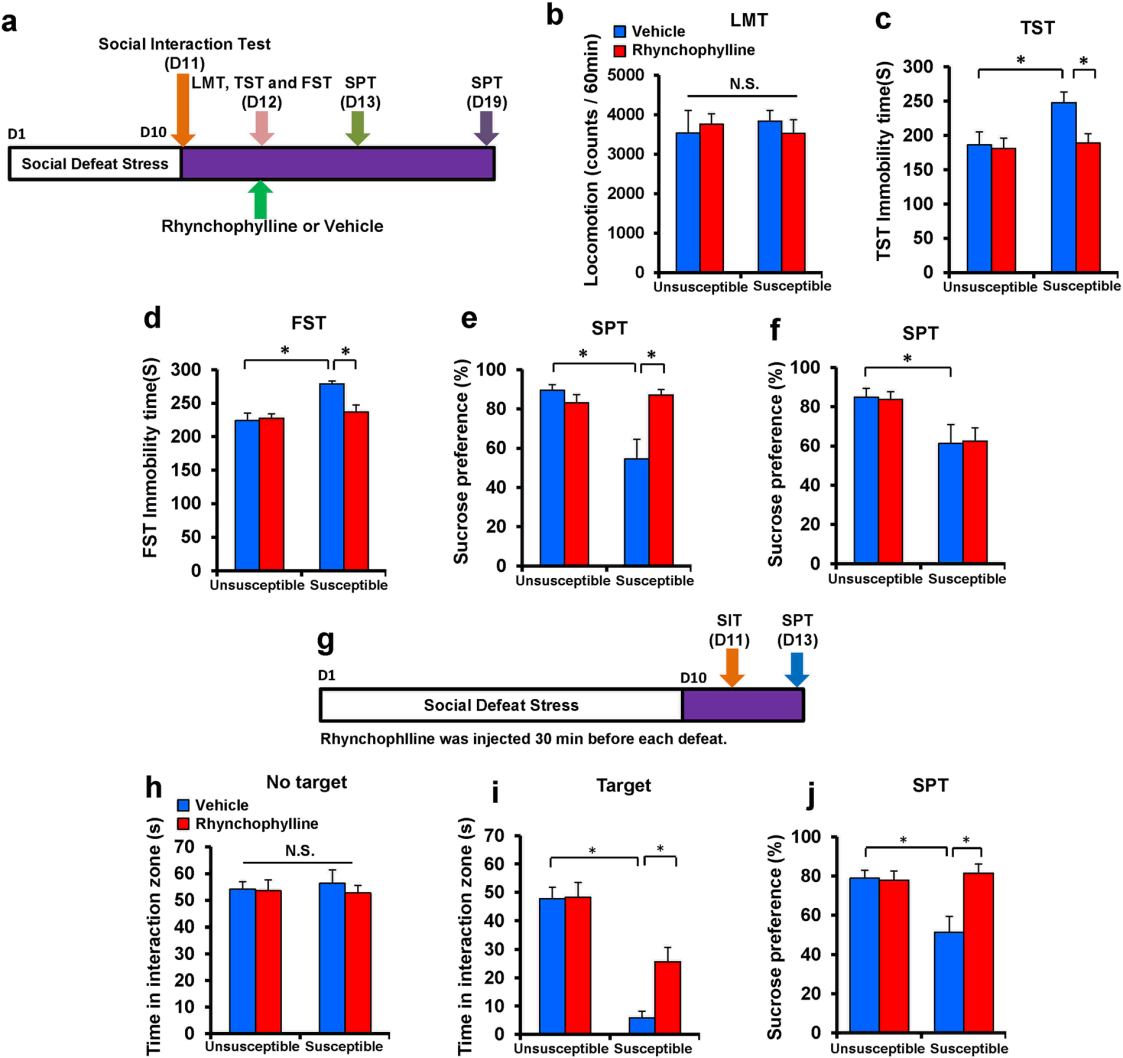
Rhynchophylline had antidepressant and prophylactic effects in a social defeat stress model (8–10 weeks old). (**a**) Schedule of social defeat stress (10 days), drug treatment, and behavioral tests. (**b**) Locomotion test (LMT). (**c**) Tail suspension test (TST). (**d**) Forced swimming test (FST). (**e**) 1% Sucrose preference test (SPT). (**F**) 1% SPT. The values represent the mean ± S.E.M. (n = 14 or 15). *P < 0.05, compared with the vehicle + stressed group. N.S.: not significant. (**g**) Schedule of social defeat stress (10 days), drug treatment, and behavior tests. (**h**) Social interaction test (SIT): no target. (**i**) SIT: target. (**j**) SPT. The values represent the mean ± S.E.M. (n = 8). *P < 0.05 compared with the vehicle + stress group. N.S.: not significant.

First, we examined the therapeutic effects of rhynchophylline on depression-like behavior in adolescent (8–10 weeks old) mice after social defeat stress. Rhynchophylline (25 mg/kg) was administered intraperitoneally to susceptible mice after social defeat stress (Fig. 2a). To examine the therapeutic effects of rhynchophylline on depression-like behavior after social defeat stress, rhynchophylline (25 mg/kg) was administered intraperitoneally to susceptible mice (8–10 weeks old) (Fig. 2a). No effect was observed in spontaneous locomotion in the four groups (Two-way ANOVA, stress: F_1,58_ = 0.008, P = 0.929, treatment: F_1,58_ = 0.014, P = 0.906, interaction: F_1,58_ = 0.571, P = 0.453) (Fig. 2b). In the tail suspension test (TST) and forced swimming test (FST), rhynchophylline significantly attenuated the increased immobility time observed in susceptible mice (two-way ANOVA, TST: stress: F_1,58_ = 4.790, P = 0.033, treatment: F_1,58_ = 4.039, P = 0.049, interaction: F_1,58_ = 2.842, P = 0.098, FST: stress: F_1,58_ = 15.562, P < 0.001, treatment: F_1,58_ = 4.961, P = 0.030, interaction: F_1,58_ = 7.865, P = 0.007) (Fig. 2c and d). In the 1% sucrose preference test (SPT), rhynchophylline significantly attenuated the decreased sucrose preference in susceptible mice 1 day after a single dose (two-way ANOVA, stress: F_1,58_ = 7.348, P = 0.009, treatment: F_1,58_ = 6.735, P = 0.012, interaction: F_1,58_ = 14.231, P < 0.001) (Fig. 2e). However, rhynchophylline did not affect the decreased sucrose preference in susceptible mice 7 days after a single dose (two-way ANOVA, stress: F_1,58_ = 11.718, P = 0.001, treatment: F_1,58_ = 0.000, P = 0.995, interaction: F_1,58_ = 0.030, P = 0.864) (Fig. 2f).

Next, we examined whether pretreatment with rhynchophylline could prevent the depression-like phenotype after repeated social defeat stress (Fig. 2g). In the social interaction test (no target), the social interaction time did not change significantly among the four groups (Fig. 2h). In the social interaction test (target), pretreatment with rhynchophylline significantly attenuated the decreased social avoidance time in stressed mice (two-way ANOVA, stress: F_1,31_ = 55.42, P < 0.001, treatment: F_1,31_ = 5.463, P = 0.027, interaction: F_1,31_ = 4.936, P = 0.035) (Fig. 2i). In the SPT, pretreatment with rhynchophylline significantly attenuated the decreased sucrose preference of stressed mice (Two-way ANOVA, stress: F_1,30_ = 5.036, P = 0.033, treatment: F_1,30_ = 7.492, P = 0.011, interaction: F_1,30_ = 8.580, P = 0.007) (Fig. 2j). These findings suggest that pretreatment with rhynchophylline confers resilience to social defeat stress.

**Figure 3:**
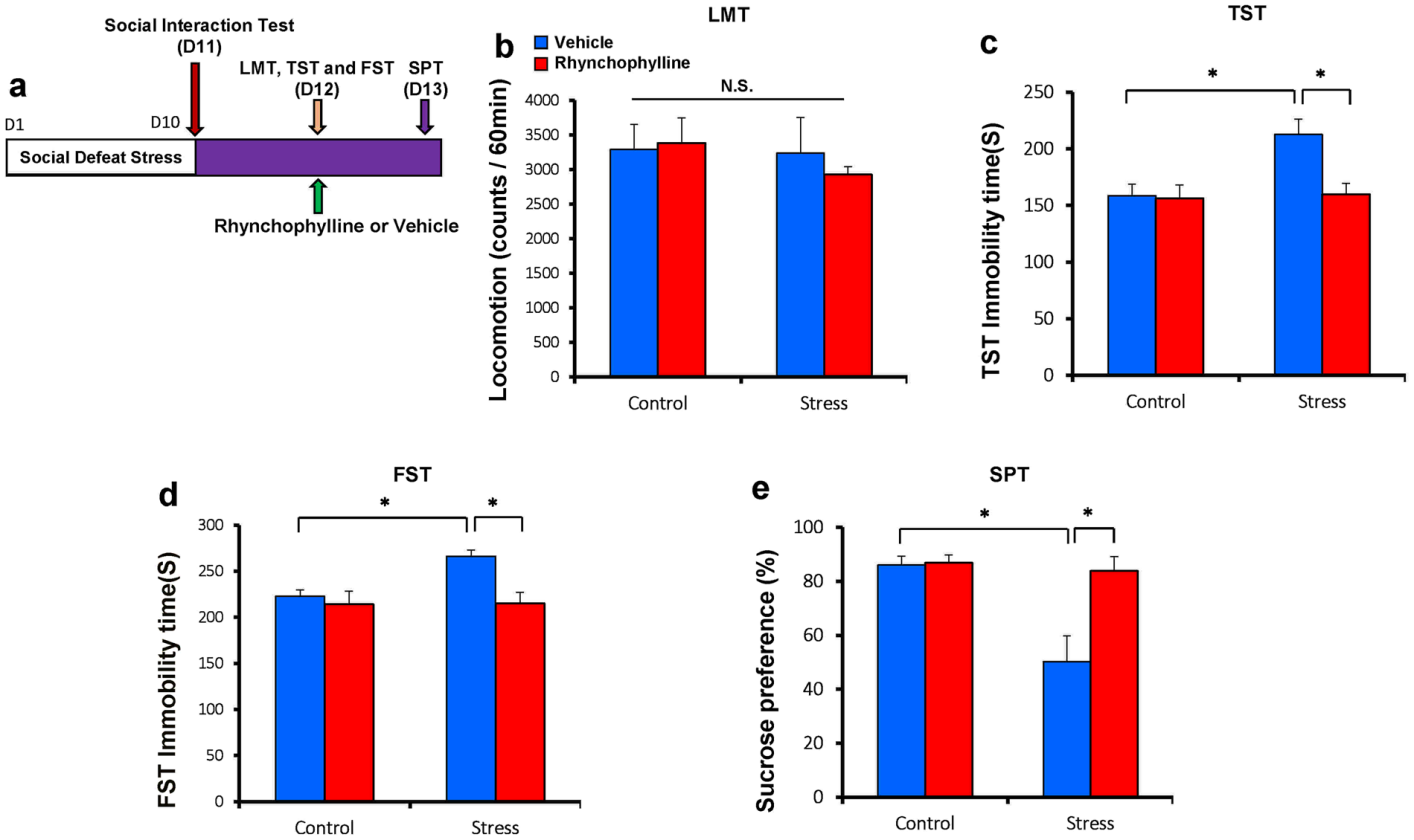
Rhynchophylline had antidepressant and prophylactic effects in a social defeat stress model (10–12 weeks old). (**a**) Schedule of social defeat stress (10 days), drug treatment, and behavioral tests. (**b**) Locomotion test (LMT). (**c**) Tail suspension test (TST). (**d**) Forced swimming test (FST). (**e**) 1% Sucrose preference test (SPT). (**F**) 1% SPT. The values represent the mean ± S.E.M. (n = 8). *P < 0.05, compared with the vehicle + stressed group. N.S.: not significant.

Finally, we examined the antidepressant-like effect of rhynchophylline in adult mice (10–12 weeks old) after social defeat stress. Rhynchophylline (25 mg/kg) was administered intraperitoneally to susceptible mice after social defeat stress (Fig. 3a). Rhynchophylline significantly attenuated the increased immobility time observed in susceptible mice in TST and FST (two-way ANOVA, TST: stress: F_1,31_ = 6.297, P = 0.018, treatment: F_1,31_ = 5.764, P = 0.024, interaction: F_1,31_ = 4.782, P = 0.038, FST: stress: F_1,31_ = 4.545, P = 0.042, treatment: F_1,31_ = 8.162, P = 0.008, interaction: F_1,31_ = 4.114, P = 0.053) (Fig. 3c and d). Furthermore, rhynchophylline significantly attenuated the decreased 0.1% SPT in susceptible mice 1 day after injection (two-way ANOVA, stress: F_1,31_ = 10.225, P = 0.004, treatment: F_1,31_ = 8.083, P = 0.009, interaction: F_1,31_ = 6.224, P = 0.019) (Fig. 3e). These results suggest that rhynchophylline showed rapid antidepressant-like effects in susceptible mice after social defeat stress.

### Antidepressant effects of rhynchophylline are in part mediated by changes in the EphA4-ephexin1-Cdk5 signaling, brain-derived neurotrophic factor (BDNF)-tropomyosin receptor kinase (TrkB) signaling and postsynaptic density protein (PSD)-95

**Figure 4:**
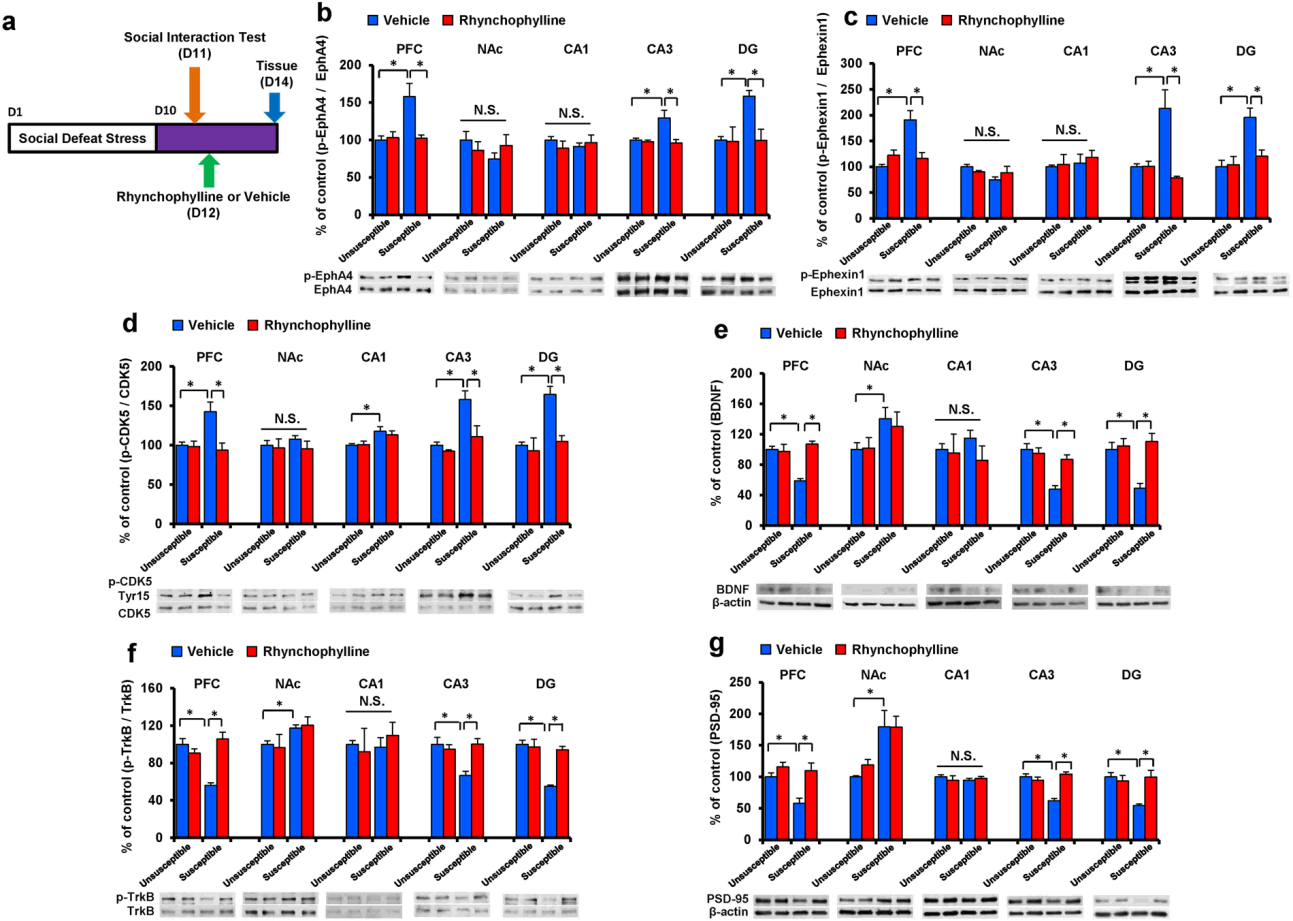
Role of EphA4-ephexin1-Cdk5 signaling, BDNF-TrkB signaling and synaptic protein in the antidepressant effect of rhynchophylline. (**a**) Schedule of social defeat stress (10 days), drug treatment, and brain sample collection. (**b**) Western blot analysis of EphA4 and p-EphA4 in the PFC, NAc, CA1, CA3, and DG of hippocampus. (**c**) Western blot analysis of ephexin1 and p-ephexin1 in the PFC, NAc, CA1, CA3, and DG of hippocampus. (**d**) Western blot analysis of p-Cdk5 (Tyr15)/Cdk5 in the PFC, NAc, CA1, CA3, and DG of hippocampus. The values are expressed as percentages relative to those in the control mice. The values represent the mean ± S.E.M. (n = 5 or 6). *P < 0.05 compared with the vehicle + stressed group. N.S.: not significant. (**e**): Western blot of BDNF in the PFC, NAc, CA1, CA3, and DG of hippocampus. (**f**): Western blot analysis of TrkB and p-TrkB in the PFC, NAc, CA1, CA3, and DG of hippocampus. (**g**): Western blot analysis of PSD-95 in the PFC, NAc, CA1, CA3, and DG of hippocampus. The values are expressed as percentages relative to those in the control mice. The values represent the mean ± S.E.M. (n = 5 or 6). *P < 0.05 compared with the vehicle + stressed group. N.S.: not significant.

We examined the molecular mechanism underlying the antidepressant-like effect of rhynchophylline (Fig. 4a–g). Social defeat stress significantly attenuated the increased p-EphA4/EphA4 and p-ephexin1/ephexin1 ratios in the PFC, CA3, and DG, but not in the CA1 and NAc, of susceptible mice. In the susceptible mice, rhynchophylline (25 mg/kg) significantly attenuated the increased p-EphA4/EphA4 and p-ephexin1/ephexin1 ratios in the PFC, CA3, and DG, but not in the CA1 and NAc (two-way ANOVA, p-EphA4/EphA4 ratio: PFC: stress: F_1,23_ = 7.655, P = 0.012, treatment: F_1,23_ = 6.514, P = 0.019, interaction: F_1,23_ = 8.187, P = 0.010, NAc: stress: F_1,23_ = 0.690, P = 0.416, treatment: F_1,23_ = 0.040, P = 0.843, interaction: F_1,23_ = 1.918, P = 0.181, CA1: stress: F_1,23_ = 0.003, P = 0.955, treatment: F_1,23_ = 0.151, P = 0.702, interaction: F_1,23_ = 1.135, P = 0.299, CA3: stress: F_1,23_ = 5.516, P = 0.029, treatment: F_1,23_ = 9.146, P = 0.007, interaction: F_1,23_ = 6.834, P = 0.017, DG: stress: F_1,23_ = 5.440, P = 0.030, treatment: F_1,23_ = 5.611, P = 0.028, interaction: F_1,23_ = 4.911, P = 0.038) (two-way ANOVA, p-ephexin1/ephexin1 ratio: PFC: stress: F_1,23_ = 12.266, P = 0.003, treatment: F_1,23_ = 4.657, P = 0.045, interaction: F_1,23_ = 16.112, P = 0.001, NAc: stress: F_1,23_ = 3.632, P = 0.072, treatment: F_1,23_ = 0.091, P = 0.767, interaction: F_1,23_ = 2.886, P = 0.106, CA1: stress: F_1,23_ = 0.513, P = 0.482, treatment: F_1,23_ = 0.294, P = 0.594, interaction: F_1,23_ = 0.049, P = 0.826, CA3: stress: F_1,23_ = 4.566, P = 0.047, treatment: F_1,23_ = 10.004, P = 0.005, interaction: F_1,23_ = 10.277, P = 0.005, DG: stress: F_1,23_ = 12.725, P = 0.002, treatment: F_1,23_ = 5.095, P = 0.037, interaction: F_1,23_ = 6.295, P = 0.022) (Fig. 4b,c). Furthermore, social defeat stress significantly increased the levels of p-Fyn/Fyn ratio in the PFC, CA3, and DG, but not in the CA1 and NAc, of susceptible mice. Rhynchophylline significantly attenuated the increased p-Fyn/Fyn ratio in the PFC, CA3, and DG, but not in the CA1 and NAc, of susceptible mice (Figure S1).

Cyclin-dependent kinase 5 (Cdk5) regulates the EphA4-mediated dendritic spine retraction via an ephexin1-dependent mechanism^20^, so we also performed Western blot for Cdk5 and its phosphorylated form p-Cdk5 (Tyr 15) in these brain regions. In susceptible mice, social defeat stress significantly increased the p-Cdk5 (Tyr 15)/Cdk5 ratios in the PFC, CA1, CA3, and DG, but not in the NAc. Rhynchophylline (25 mg/kg) significantly attenuated the increased p-Cdk5 (Tyr 15)/Cdk5 ratio in the PFC, CA3, and DG, but not in the NAc and CA1, of susceptible mice (two-way ANOVA, p-Cdk5 (Tyr 15)/Cdk5 ratio: PFC: stress: F_1,22_ = 4.578, P = 0.046, treatment: F_1,22_ = 8.138, P = 0.011, interaction: F_1,22_ = 7.108, P = 0.016, NAc: stress: F_1,23_ = 0.120, P = 0.733, treatment: F_1,23_ = 0.763, P = 0.394, interaction: F_1,23_ = 0.231, P = 0.636, CA1: stress: F_1,23_ = 10.112, P = 0.005, treatment: F_1,23_ = 0.173, P = 0.683, interaction: F_1,23_ = 0.306, P = 0.587, CA3: stress: F_1,23_ = 15.612, P = 0.001, treatment: F_1,23_ = 8.076, P = 0.011, interaction: F_1,23_ = 4.226, P = 0.055, DG: stress: F_1,23_ = 12.666, P = 0.002, treatment: F_1,23_ = 9.684, P = 0.006, interaction: F_1,23_ = 5.959, P = 0.025) (Fig. 4d). These results suggest that rhynchophylline significantly attenuated the increased ratios of p-EphA4/EphA4, p-ephexin1/ephexin1 and p-Cdk5 (Tyr 15)/Cdk5 in the PFC, CA3, and DG of susceptible mice after social defeat stress.

Signaling by BDNF and its receptor TrkB plays a key role in the depression-like phenotype^30–36^, so we examined the role of BDNF-TrkB signaling in the selected brain regions after rhynchophylline treatment. In susceptible mice, social defeat stress significantly decreased the levels of BDNF and p-TrkB/TrkB ratio in the PFC, CA3, and DG, but not in the NAc and CA1. A single administration of rhynchophylline (25 mg/kg) significantly attenuated the decreased levels of BDNF and the p-TrkB/TrkB ratio in the PFC, CA3, and DG, but not in the NAc and CA1 (two-way ANOVA, BDNF: PFC: stress: F_1,21_ = 7.606, P = 0.013, treatment: F_1,21_ = 15.908, P = 0.001, interaction: F_1,21_ = 19.563, P < 0.001, NAc: stress: F_1,21_ = 5.236, P = 0.034, treatment: F_1,21_ = 0.083, P = 0.776, interaction: F_1,21_ = 0.161, P = 0.693, CA1: stress: F_1,21_ = 0.026, P = 0.873, treatment: F_1,21_ = 0.993, P = 0.332, interaction: F_1,21_ = 0.507, P = 0.486, CA3: stress: F_1,21_ = 20.159, P < 0.001, treatment: F_1,21_ = 6.404, P = 0.021, interaction: F_1,21_ = 11.004, P = 0.004, DG: stress: F_1,21_ = 5.567, P = 0.030, treatment: F_1,21_ = 11.875, P = 0.003, interaction: F_1,21_ = 8.775, P = 0.008)(two-way ANOVA, p-TrkB/TrkB ratio: PFC: stress: F_1,21_ = 6.452, P = 0.021, treatment: F_1,21_ = 12.494, P = 0.002, interaction: F_1,21_ = 27.140, P < 0.001, NAc: stress: F_1,21_ = 5.676, P = 0.028, treatment: F_1,21_ = 0.001, P = 0.982, interaction: F_1,21_ = 0.144, P = 0.709, CA1: stress: F_1,21_ = 0.535, P = 0.474, treatment: F_1,21_ = 0.056, P = 0.815, interaction: F_1,21_ = 1.107, P = 0.307, CA3: stress: F_1,21_ = 5.262, P = 0.034, treatment: F_1,21_ = 5.338, P = 0.033, interaction: F_1,21_ = 9.832, P = 0.006; DG: stress: F_1,21_ = 22.324, P < 0.001, treatment: F_1,21_ = 12.841, P = 0.002, interaction: F_1,21_ = 16.971, P = 0.001) (Fig. 4e and f). Furthermore, we performed Western blot for the synaptogenesis marker PSD-95. In susceptible mice, social defeat stress significantly decreased the levels of PSD-95 in the PFC, CA3, and DG, but not in the NAc and CA1 (Fig. 4g). Rhynchophylline (25 mg/kg) significantly attenuated the decreased levels of PSD-95 in the PFC, CA3, and DG, but not in the NAc and CA1 of susceptible mice (two-way ANOVA, PSD-95: PFC: stress: F_1,22_ = 7.028, P = 0.016, treatment: F_1,22_ = 14.169, P = 0.001, interaction: F_1,22_ = 4.04, P = 0.059, NAc: stress: F_1,22_ = 16.752, P = 0.001, treatment: F_1,22_ = 0.295, P = 0.594, interaction: F_1,22_ = 0.322, P = 0.577, CA1: stress: F_1,22_ = 0.071, P = 0.792, treatment: F_1,22_ = 0.092, P = 0.765, interaction: F_1,22_ = 0.949, P = 0.342, CA3: stress: F_1,22_ = 9.993, P = 0.005, treatment: F_1,22_ = 16.943, P = 0.001, interaction: F_1,22_ = 28.544, P < 0.001, DG: stress: F_1,22_ = 6.264, P = 0.022, treatment: F_1,22_ = 5.980, P = 0.025, interaction: F_1,22_ = 10.797, P = 0.004) (Fig. 4g). These results suggest that rhynchophylline shows an antidepressant-like effect by activating BDNF-TrkB signaling and synaptogenesis in the PFC, CA3, and DG in mice with the depression-like phenotype.

### Effect of rhynchophylline on alterations in the dendritic spine density induced by social defeat stress

**Figure 5:**
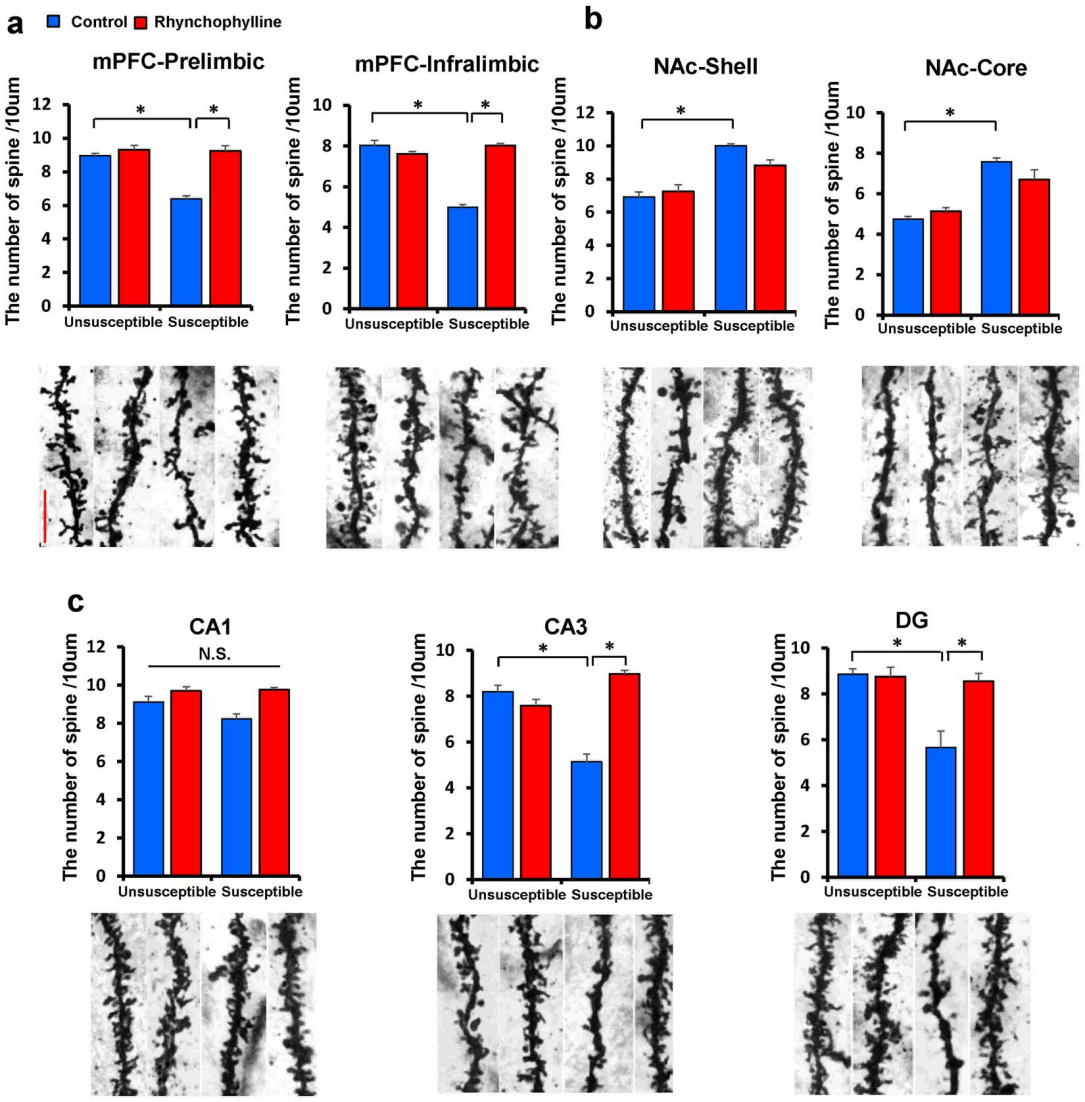
Effects of rhynchophylline on stress-induced changes in the spine density within brain. (**a**,**b** and **c**) Representative photomicrographs of Golgi-Cox stained pyramidal neurons in the prelimbic and infralimbic of mPFC, NAc-shell and NAc-core, CA1, CA3 and DG of the hippocampus, from each group. Scale bar = 10 μm. The values represent the mean ± S.E.M. (n = 6). *P < 0.05 compared with the control + stressed group. N.S.: not significant.

Alterations in the dendritic length and spine density in the PFC, NAc and hippocampus play an important role in the pathophysiology of depression, and antidepressant treatment can block or revers these changes^30, 37, 38^. Therefore, we examined whether rhynchophylline showed antidepressant-like effects by changing the dendritic spines of the PFC, NAc and hippocampus. Social defeat stress decreased the spine density in the medial PFC (mPFC prelimbic and infralimbic regions) and hippocampus (CA3 and DG), whereas the stress increased the spine density in NAc (shell and core) (Fig. 5a,b and c). Treatment with rhynchophylline significantly attenuated the stress-induced reduction of spine density in the mPFC (prelimbic and infralimbic) and hippocampus (CA3 and DG) (two-way ANOVA, prelimbic: stress: F_1,23_ = 27.611, P < 0.001, treatment: F_1,23_ = 41.087, P < 0.001, interaction: F_1,23_ = 25.325, P < 0.001, infralimbic: stress: F_1,23_ = 68.816, P < 0.001, treatment: F_1,23_ = 67.116, P < 0.001, interaction: F_1,23_ = 119.751, P < 0.001, CA3: stress: F_1,23_ = 9.375, P = 0.006, treatment: F_1,23_ = 35.042, P < 0.001, interaction: F_1,23_ = 66.634, P < 0.001, DG: stress: F_1,23_ = 60.098, P < 0.001, treatment: F_1,23_ = 42.512, P < 0.001, interaction: F_1,23_ = 66.634, P < 0.001) (Fig. 5a and c). However, treatment with rhynchophylline did not attenuate stress-induced increases of spine density in the NAc (shell and core) (two-way ANOVA, shell: stress: F_1,23_ = 37.575, P < 0.001, treatment: F_1,23_ = 0.240, P = 0.629, interaction: F_1,23_ = 2.164, P = 0.157, core: stress: F_1,23_ = 62.535, P < 0.001, treatment: F_1,23_ = 0.812, P = 0.378, interaction: F_1,23_ = 5.301, P = 0.032) (Fig. 5b). These results suggest that rhynchophylline showed antidepressant-like effects by normalizing the reduced spine density in the brain regions (mPFC, CA3, DG) after social defeat stress.

### Overexpression of EphA4 into the mPFC produced depression-like behavior

**Figure 6:**
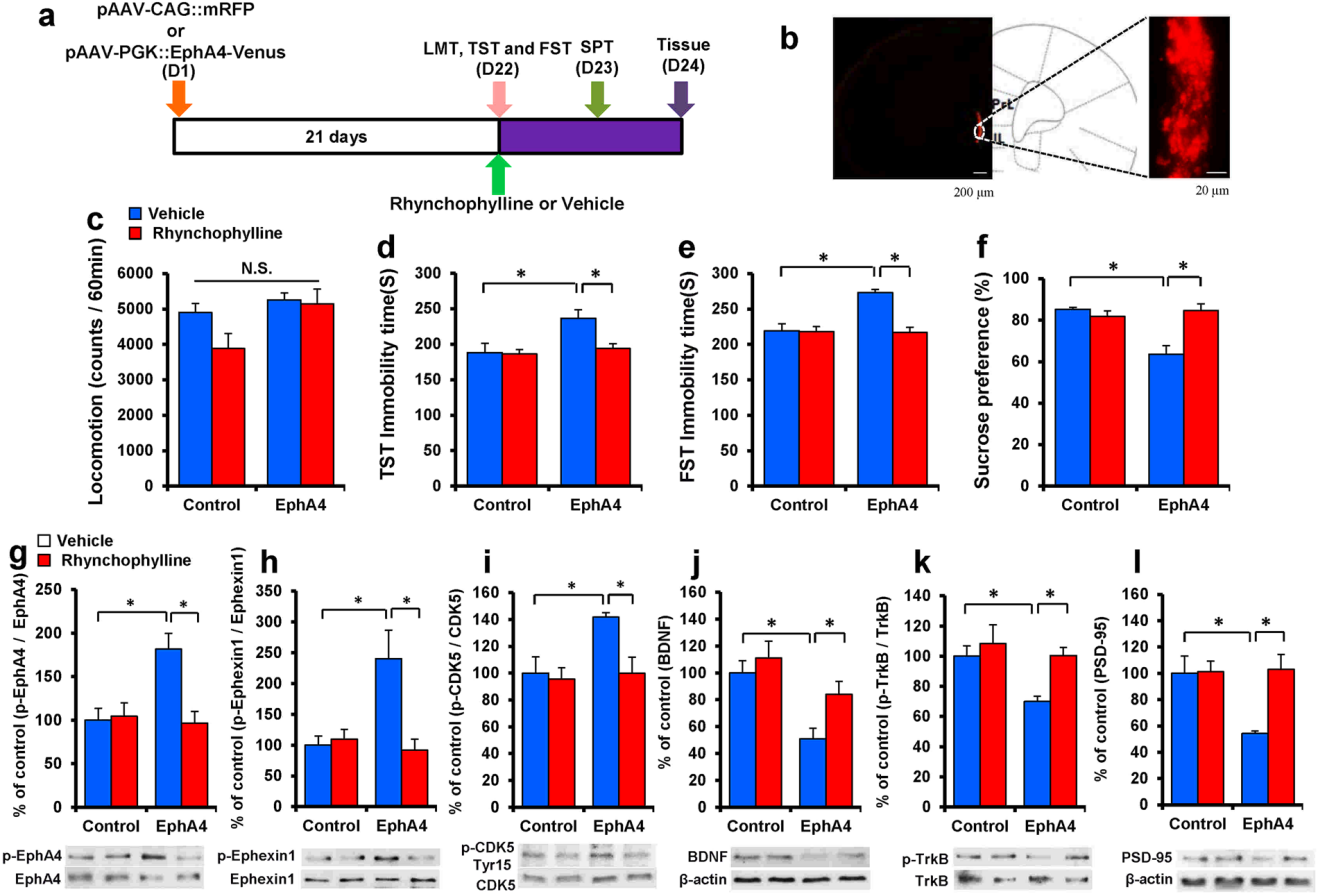
Depression-like phenotype after bilateral injection of pAAV-PGK:: EphA4-Venus into the mPFC. (**a**) Schedule of pAAV vector injection, drug treatment, and behavioral tests. (**b**) Representative photographs of the injection sites and coronal brain sections in the mPFC. The success of AAV vector injection into the mPFC was confirmed by the presence of mRFP fluorescence. Scale bars = 200 μm (low-power images) and 20 μm (high-power images). (**c**) LMT. (**d**) TST. (**e**) FST. (**f**) SPT. The values represent the mean ± S.E.M. (n = 7). *P < 0.05 compared with the vehicle + EphA4 group. N.S.: not significant. (**g**) p-EphA4/EphA4 ratio in the PFC. (**h**) p-Ephexin1/Ephexin1 ratio in the PFC. (**i**) p-Cdk5(Tyr15)/Cdk5 ratio in the PFC. (**j**) BDNF in the PFC. (**k**) p-TrkB/TrkB ratio in the PFC. (l) PSD-95 in the PFC. The values represent the mean ± S.E.M. (n = 5 or 6). *P < 0.05 compared with the vehicle + EphA4 group. N.S.: not significant.

We examined whether the overexpression of EphA4 in the mPFC after the administration of EphA4 AAV could induce depression-like behavior in mice (Fig. 6a and b). There were no changes in spontaneous locomotion among the four groups (two-way ANOVA, AAV: F_1,27_ = 3.083, P = 0.092, treatment: F_1,27_ = 1.527, P = 0.229, interaction: F_1,27_ = 0.981, P = 0.332) (Fig. 6c). In the TST and FST, the immobility time was increased significantly 3 weeks after bilateral injection of EphA4 AAV into the mPFC. Rhynchophylline (25 mg/kg) significantly attenuated the increased immobility time observed in mice after the administration of EphA4 AAV (two-way ANOVA, TST: AAV: F_1,27_ = 24.522, P < 0.001, treatment: F_1,27_ = 6.540, P = 0.017, interaction: F_1,27_ = 17.945, P < 0.001, FST: AAV: F_1,27_ = 6.449, P = 0.018, treatment: F_1,27_ = 7.623, P = 0.011, interaction: F_1,27_ = 6.926, P = 0.015)(Fig. 6d and e). Furthermore, rhynchophylline significantly attenuated the decreased sucrose preference observed in mice after treatment with EphA4 AAV (two-way ANOVA, AAV: F_1,27_ = 4.928, P = 0.036, treatment: F_1,27_ = 4.396, P = 0.047, interaction: F_1,27_ = 8.406, P = 0.008) (Fig. 6f).

Next, we examined the p-EphA4/EphA4, p-ephexin1/ephexin1, and p-Cdk5 (Tyr-15)/Cdk5 ratios in the PFC. The administration of EphA4 AAV significantly increased the p-EphA4/EphA4, p-ephexin1/ephexin1, and p-Cdk5 (Tyr-15)/Cdk5 ratios in the PFC (Fig. 6g–i). Rhynchophylline (25 mg/kg) significantly attenuated the increased p-EphA4/EphA4, p-ephexin1/ephexin1, and p-Cdk5 (Tyr 15)/Cdk5 ratios in the PFC (two-way ANOVA, p-EphA4/EphA4 ratio: AAV: F_1,22_ = 5.766, P = 0.027, treatment: F_1,22_ = 6.834, P = 0.017, interaction: F_1,22_ = 8.441, P = 0.009; p-Ephexin1/Ephexin1 ratio: AAV: F_1,22_ = 5.462, P = 0.031, treatment: F_1,22_ = 7.017, P = 0.016, interaction: F_1,22_ = 9.119, P = 0.007, p-Cdk5/Cdk5 ratio, AAV: F_1,23_ = 5.067, P = 0.036, treatment: F_1,23_ = 5.118, P = 0.036, interaction: F_1,23_ = 3.372, P = 0.082) (Fig. 6g–i). Furthermore, the administration of EphA4 AAV significantly increased the p-Fyn/Fyn ratio, and rhynchophylline (25 mg/kg) significantly attenuated the increased p-Fyn/Fyn ratio in the PFC (Figure S2).

Moreover, the administration of EphA4 AAV significantly decreased the expression of BDNF, the p-TrkB/TrkB ratio, and the PSD-95 level in the PFC (Fig. 6j–l). The administration of rhynchophylline (25 mg/kg) also significantly attenuated the decreased levels of BDNF and PSD-95, and the p-TrkB/TrkB ratio in the PFC (two-way ANOVA, BDNF: AAV: F_1,22_ = 13.995, P = 0.001, treatment: F_1,22_ = 4.671, P = 0.044, interaction: F_1,22_ = 1.172, P = 0.292; p-TrkB/TrkB ratio: AAV: F_1,22_ = 5.449, P = 0.031, treatment: F_1,22_ = 5.684, P = 0.028, interaction: F_1,22_ = 1.83, P = 0.192, PSD-95: AAV: F_1,22_ = 4.451, P = 0.049, treatment: F_1,22_ = 5.749, P = 0.028, interaction: F_1,22_ = 5.209, P = 0.035) (Fig. 6j–l). These results suggest that the administration of EphA4 AAV into the mPFC activated EphA4–ephexin1 signaling and the subsequent inhibition of BDNF-TrkB signaling in the PFC, thereby resulting in the depression-like phenotype. Rhynchophylline showed a rapid antidepressant-like effect by blocking EphA4-ephexin1 signaling in the PFC.

## Discussion

In the present study, we demonstrated a key role for EphA4-ephexin1 signaling in the pathophysiology of depression and our main findings are as follows. First, we found that EphA4-ephexin1 signaling was activated in the PFC, CA3, and DG of mice with the depression-like phenotype. Furthermore, the p-EphA4/EphA4 ratio was higher in the parietal cortex from depressed patients compared with the controls. Second, a novel EphA4 inhibitor, rhynchophylline, had a rapid antidepressant-like effect and prophylactic effects in a social defeat stress model. Finally, a bilateral injection of EphA4 AAV into the mPFC produced the depression-like phenotype in mice. Interestingly, rhynchophylline had a rapid antidepressant-like effect by inhibiting increased EphA4-ephexin1 signaling in the mPFC. In addition, rhynchophylline showed a rapid antidepressant-like effect in susceptible mice at 10 weeks old and 12 weeks old, suggesting an antidepressant-like effect of rhynchophylline in late adolescent and adult mice. These findings suggest that the activation of EphA4-ephexin1 signaling in the PFC plays a key role in the pathophysiology of depression, thereby indicating that EphA4-ephexin1 signaling could be a novel therapeutic target for depression.

EphA4 is expressed in the PFC and hippocampus of the adult brain, where it acts as a negative regulator of NMDA receptor neurotransmission^14^. EphA4 activation by ephrins triggers forward signaling, which leads to the retraction of dendritic spines via the activation of ephexin1^12, 19–21^. EphA4 also causes the removal of synaptic and surface α-amino-3-hydroxy-5-methyl-4-isoxazolepropionic acid (AMPA) receptors during homeostatic plasticity^18^. It is well known that alterations in the dendritic spine morphology in the PFC and hippocampus play a role in the neurobiology of depression^22–24^. In this study, rhynchophylline produced a rapid antidepressant-like effect by normalizing increased EphA4-ephexin1 signaling in the PFC and hippocampus of susceptible mice and by normalizing the decreased spine density in the PFC and hippocampus of susceptible mice. Furthermore, we found an increased EphA4 signaling in the parietal cortex from depressed patients. In contrast, Li *et al*.^39^ reported decreased expression of EphA4 in the hippocampus from rats with depression-like phenotype in the chronic mild stress model although the p-EphA4/EphA4 ratio was not measured. We also found increased p-EphA4/EphA4 and p-ephexin1/ephexin1 ratios in the PFC and hippocampus of LH rats compared with the control rats, but not in the non-LH (resilient) rats. EphA4-ephexin1 signaling was activated in the susceptible LH rats, so it is possible that EphA4-ephexin1 signaling in the PFC and hippocampus may partly contribute to stress resilience. Given the role of EphA4-ephexin1 signaling in the dendritic spine morphology^25, 40^, these findings suggest that EphA4-ephexin1 signaling might play a key role in the pathophysiology of depression, and the antidepressant-like effect of rhynchophylline might be at least in part due to an impairment of ephrin reverse signaling.

Accumulating evidence suggests that Cdk5 also plays an important role in the pathophysiology of depression. It has been reported that stress can increase the Cdk5 levels in the PFC and hippocampus of rodents^41, 42^. In contrast, loss of Cdk5 function in the ventral tegmental area (VTA) induced anxiety- and depression-like behaviors^43^. A recent study demonstrated that the overexpression of Cdk5 by Cdk5-ZFP1-p65 in the NAc of mice could make mice more resilient to social defeat stress^44^. These findings suggest that abnormal Cdk5 levels in the PFC and hippocampus, as well as the VTA-NAc pathway might have causative roles in depression. In this study, we found that the p-Cdk5 (Tyr 15)/Cdk5 ratio increased in the PFC and hippocampus of mice with the depression-like phenotype, but not in the NAc. Interestingly, rhynchophylline significantly attenuated the increased p-Cdk5 (Tyr 15)/Cdk5 ratio in the PFC and hippocampus, thereby suggesting that Cdk5 might play a role in the antidepressant-like effect of rhynchophylline in the PFC and hippocampus.

It is well known that BDNF-TrkB signaling plays a key role in depression^30–36^. Previous studies of postmortem brains showed that the BDNF levels were decreased in the PFC and hippocampus of psychiatric disorder patients who had committed suicide compared with non-psychiatric controls^45, 46^, which suggests that the decreased BDNF levels in the PFC and hippocampus may have causative roles in the pathophysiology of depression. We found decreased levels of BDNF in the PFC and hippocampus of rodents with the depression-like phenotype^37, 38, 47–49^. Similar to the NMDA receptor antagonist ketamine^47, 50^, rhynchophylline produced a rapid antidepressant-like effect by normalizing decreased BDNF-TrkB signaling in the PFC and hippocampus of mice with depression-like phenotype. However, unlike ketamine, rhynchophylline did not have a sustained antidepressant-like effect.

The previous studies demonstrated that increased BDNF-TrkB signaling in the VTA-NAc pathway plays key a role in the depression-like phenotype^30, 31, 37, 38, 45, 47^. A study using postmortem brain samples showed increased levels of BDNF in the NAc of patients with depression relative to controls^51^. We reported that lipopolysaccharide-induced inflammation and social defeat stress increased BDNF levels in the NAc, resulting in depression-like phenotype in mice. Interestingly, ANA-12, a TrkB antagonist, showed an antidepressant-like effect by blocking TrkB in the NAc^30, 37, 47, 52, 53^. Therefore, increased BDNF–TrkB signaling in NAc may play a causative role in the pathophysiology of depression. However, rhynchophylline did not alter BDNF, p-TrkB/TrkB ratio and PSD-95 in the NAc. Taken together, it is likely that rhynchophylline could show antidepressant-like effect by activating BDNF-TrkB signaling in the PFC and hippocampus, but not in NAc, of susceptible mice. However, the precise mechanisms underlying the relationship between EphA4 signaling and BDNF-TrkB signaling are currently unknown.

**Figure 7:**
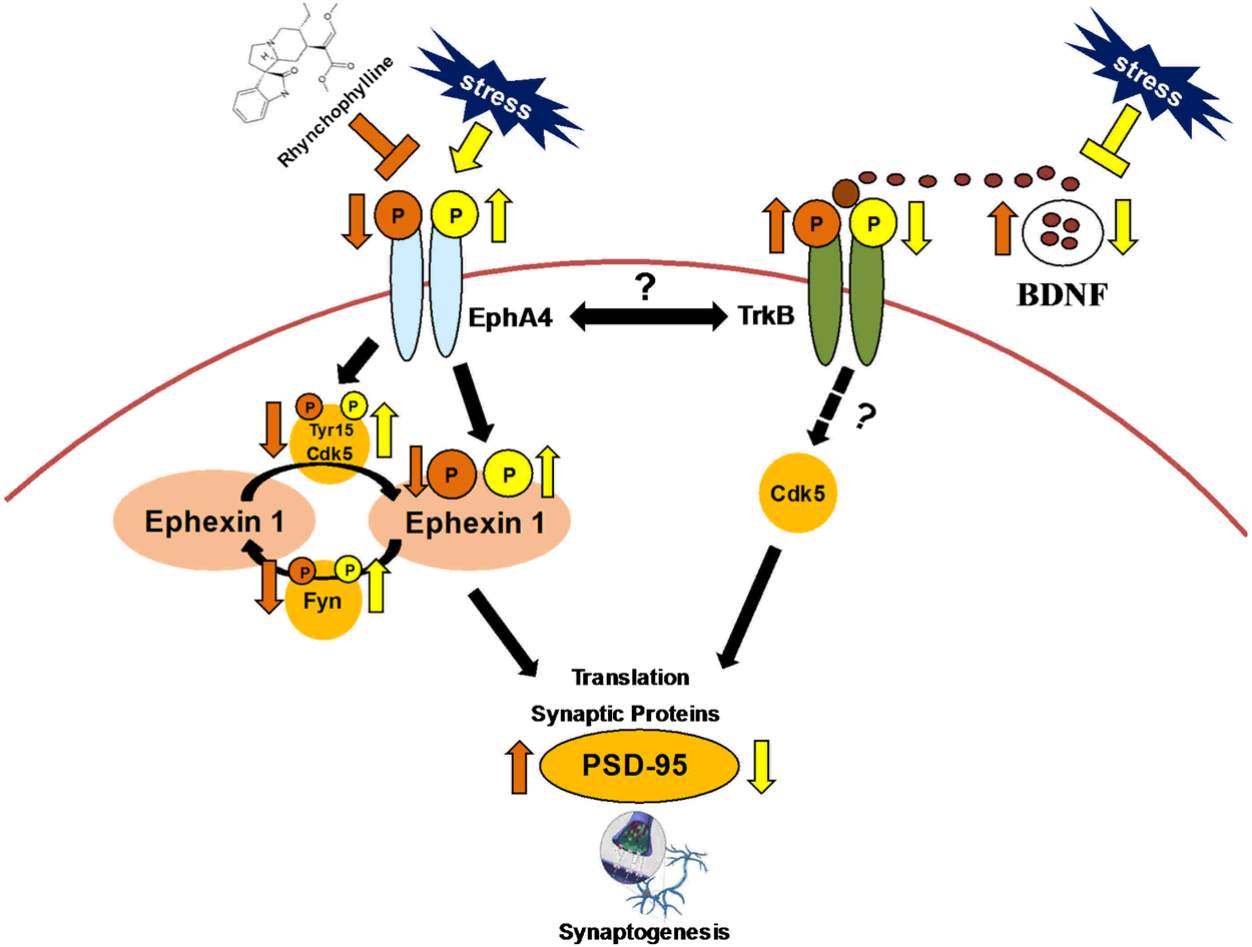
Working model of the EphA4–ephexin1 signaling pathway and BDNF-TrkB signaling pathway in the model of depression. Social defeat stress promotes the activation of EphA4–ephexin1 signaling, which mediates synaptic loss. The activation of EphA4 by stress enhances the activity of Cdk5 via the phosphorylation of Cdk5 at Tyr15 and ephexin1. The activation of Cdk5 at Tyr15 and Fyn promotes ephexin1 phosphorylation, thereby leading to decreased synaptogenesis and dendrite atrophy. Social defeat stress inhibits BDNF-TrkB signaling, which could mediate synaptic loss. Rhynchophylline attenuates the activation of EphA4–ephexin1 signaling and the inhibition of BDNF-TrkB signaling in mice with the depression-like phenotype. The inhibition of EphA4–ephexin1 signaling can improve the decreased BDNF-TrkB signaling in depression, thereby improving the synaptic protein level. The inhibition of Cdk5 or absence of the Cdk5 activator p35 completely abolishes BDNF-induced spine formation and enlargement^62^, so it is likely that Cdk5 may work downstream of BDNF-TrkB signaling.

EphA4 signaling was the first extracellular cue to be identified as phosphorylating ephexin1, and Cdk5 promote its activity in central nervous system synapses^19, 20, 54^. EphA4 stimulation by an ephrin-A ligand enhances the Cdk5 activity via the phosphorylation of Cdk5 at Tyr15^19, 20^. Importantly, ephexin1, a Rho GEF, is phosphorylated by Cdk5 *in vivo*. Ephexin1 has been reported to transduce signals from activated EphA4 to RhoA, thereby resulting in growth cone collapse during axon guidance^19, 20^. The loss of ephexin1 in cultured hippocampal neurons or *in vivo* perturbs the ability of ephrin-A to induce EphA4-dependent spine retraction. Therefore, the phosphorylation of ephexin1 by Cdk5 is required for EphA4-dependent spine retraction. BDNF-TrkB signaling is also implicated in synapse and spine development. Thus, BDNF triggers serine phosphorylation of TrkB in the juxtamembrane region at S478 by Cdk5^54, 55^. Overexpression of a TrkB construct that lacks the Cdk5 phosphorylation site also inhibits neurite outgrowth by cultured hippocampal neurons in response to BDNF, which suggests that serine phosphorylation might be crucial for TrkB signaling pathway^55^. In addition, the inhibition of Cdk5 or absence of the Cdk5 activator p35 completely abolishes BDNF-induced spine formation and enlargement^55^. These results indicate that Cdk5 may be a downstream protein in the BDNF-TrkB signaling pathway. Therefore, it is likely that Cdk5 plays an important role in both the EphA4–ephexin1 and BDNF-TrkB signaling pathways. Based on the present findings, we propose a framework for the pathological changes in the PFC and hippocampus after experiencing social defeat stress and the possible therapeutic mechanism of EphA4 inhibition (Fig. 7). In the current study, we found that stress activates EphA4-ephexin1 signaling and inhibits BDNF-TrkB signaling in the PFC and hippocampus, which reduced the abundance of the synaptic protein PSD-95. Rhynchophylline could normalize increased EphA4-ephexin1 signaling and decreased BDNF-TrkB signaling in the PFC and hippocampus of susceptible mice, which resulted in its rapid antidepressant-like effect. However, the precise mechanisms that underlie the relationship between EphA4-ephexin1 signaling and BDNF-TrkB signaling are currently unknown.

Patients with depression become chronically ill, with several relapses (early return of symptoms within the expected duration of a current episode, possibly 3–12 months) or later recurrences (new episodes) following initial short-term improvement or remission^56, 57^. The recurrence rates are over 85% within a decade of an index depressive episode, with an average of approximately 50% or more within six months of apparent clinical remission^57^. Therefore, the prevention of relapse and recurrence is very important for the management of depression. In this study, we demonstrated the prophylactic effects of rhynchophylline in social defeat stress models of depression, which suggests that rhynchophylline could prevent the onset of the depression-like phenotype caused by social defeat stress. Considering the inclusion of rhynchophylline in traditional Chinese/Japanese medicine^26, 27, 58^, it is likely that rhynchophylline could be used as a natural prophylactic compound to prevent or minimize the relapses caused by stress during the remission state in depressed patients.

A previous study showed that rhynchophylline acts as non-competitive antagonist of NMDA receptor^59^. Subsequently, it is reported that rhynchophylline exerts its protective action by inhibiting NMDA, muscarinic M_1_, and serotonin 5-HT_2_ receptor-mediated neurotoxicity during ischemia^60^. These findings suggest that rhynchophylline is not a selective EphA4 inhibitor. Therefore, further detailed experiments using a selective EphA4 inhibitor will be needed.

In conclusion, these findings suggest that increased EphA4-ephexin1 signaling in the PFC and hippocampus play key roles in the depression phenotype after repeated social defeat stress in mice, and that rhynchophylline has a rapid antidepressant-like effect. Therefore, EphA4-ephexin1 signaling could be a novel therapeutic target for depression.

## Materials and Methods

### Animals and animal care

Male C57BL/6 mice (n = 182), aged 8 or 10 weeks (body weight 20–25 g; Japan SLC Inc., Hamamatsu, Japan) or 5 weeks (body weight 15–20 g; Japan SLC Inc., Hamamatsu, Japan), and male adult CD1 mice (n = 60) aged 13–15 weeks (body weight >40 g; Japan SLC, Inc., Hamamatsu, Japan) were used in the experiments. Animals were housed in polycarbonate cage (22.5 × 33.8 × 14.0 cm) under controlled temperatures with a 12 h: 12 h light: dark cycle (lights on between 07:00–19:00 h), where food (CE-2; Japan CLEA, Ltd., Tokyo, Japan) and water were available *ad libitum*. The protocol was approved by Chiba University Institutional Animal Care and Use Committee, and the Animal Experiment Committee of the University of Tokyo. This study was carried out in strict accordance with the recommendations in the Guide for the Care and Use of Laboratory Animals of the National Institutes of Health, USA. Animals were deeply anaesthetized with isoflurane before being killed by cervical dislocation. All efforts were made to minimize animal suffering.

### Drug administration

On the day of injection, fresh solutions were prepared by dissolving compounds in sterile endotoxin-free isotonic saline. The dose of rhynchophylline (25 mg/kg; Catalog number: R0105, Tokyo Chemical Industry, Tokyo, Japan) was based on a previous report^25^ and the preliminary experiment. Rhynchophylline was dissolved in saline using ultrasound. Control (unsusceptible) mice and susceptible mice were randomly divided into two groups, respectively.

### Social defeat procedure

The social defeat procedure was performed as reported previously^47, 50, 61–63^. Each day, the C57BL/6 mice (8 weeks or 10 weeks old) were exposed to a different CD1 aggressor mouse for 10 min for a total of 10 days. When the social defeat session ended, the resident CD1 mouse and the intruder mouse were housed in one half of the cage separated by a perforated Plexiglas divider to allow visual, olfactory, and auditory contact for the remainder of the 24-h period. At 24 h after the last session, all mice were housed individually. On day 11, a social avoidance test was performed to identify subgroups of mice that were susceptible and not susceptible to social defeat stress. A social interaction test was performed on the day after the last social defeat session. In this test, an open field arena (42 × 42 cm) was divided into an interaction zone and two opposing corner zones. A plastic mesh target box (10 × 4.5 cm) was placed in the interaction zone. A test mouse was allowed to roam around the open field arena for 2.5 min with no social target (CD1 mouse) in the mesh box (which is denoted as “no target” in the figures showing the results of the social-interaction experiments). Next, a novel CD1 mouse was placed in a metal/plastic mesh target box in the interaction zone (which is denoted as “target” in the figures showing the results of the social-interaction experiments) and the test mouse was placed back in the open arena for another 2.5 min. Using a stopwatch, the amount of time spent in the interaction zone (defined as the 8 cm-wide area surrounding the wire mesh cage) was measured with or without the social target during 2.5 min^63^. The interaction ratio was calculated as 100 × (time spent in an interaction zone with an aggressor)/(time spent in an interaction zone without an aggressor). An interaction ratio of 100 was set as the cutoff, where mice with scores <100 were defined as “susceptible” to social defeat stress and those with scores ≥100 were defined as “unsusceptible.” Approximately 70% of mice were susceptible in this study. The resilience mice were removed from the experiments.

### Stress paradigm: learned helplessness (LH) model

The LH paradigm and behavioral tests were performed using the Gemini Avoidance System (San Diego Instruments, San Diego, CA, USA)^37, 48, 49^. The apparatus was divided into two compartments by a retractable door. On days 1 and 2, male Sprague-Dawley rats (200–300 g; 7 weeks old, Charles River Japan Co., Tokyo, Japan) were subjected to 30 inescapable electric foot shocks (0.65 mA, 30-s duration, at random intervals averaging 18–42 s). On day 3, a two-way conditioned avoidance test was performed as a post-shock test to determine whether the rats exhibited the predicted escape deficits. This screening process comprised 30 trials where the electric foot shocks (0.65 mA, 6-s duration, at random intervals averaging 30 s) were preceded by a 3-s conditioned stimulus tone, which remained active until the shock was terminated. The numbers of escape failures and the latency to escape were recorded in each trial. Rats with more than 25 escape failures in the 30 trials were regarded as having satisfied the criterion for the depression-like phenotype (susceptible). Approximately 65% of the rats satisfied this criterion. Rats with fewer than 25 failures that did not satisfy the criterion were defined as non-LH rats (resilience). On day 8, the animals were decapitated. The left brain hemispheres were used for conducting the western blot, as reported previously^37, 48, 49^.

### Behavioral tests

The behavioral tests were performed by two observers who were blinded to the group assignment of animals. These tests were performed as follows. Locomotion test: The mice were placed in experimental cages (length × width × height: 560 × 560 × 330 mm), where the locomotor activity of mice was counted using a SCANET MV-40 system (Melquest Co. Ltd, Toyama, Japan) and the cumulative exercise was recorded for 60 min. Cages were cleaned between test sessions. Tail suspension test (TST): The mice were taken from their home cage and a small piece of adhesive tape was placed approximately 2 cm from the tip of their tail. A single hole was punched in the tape and mice were hung individually on a hook. The immobility time of each mouse was recorded for 10 min. Mice were considered immobile only when they hung passively and completely motionless. Forced swimming test (FST): The mice were placed individually in a cylinder (diameter: 23 cm; height: 31 cm) containing 15 cm of water, which was maintained at 23 ± 1 °C. Animals were tested in an automated forced-swimming apparatus using a SCANET MV-40 system (MELQUEST Co. Ltd, Toyama, Japan). The immobility time was calculated from the activity time as (total) – (active) time, using the analysis software provided with the apparatus. The cumulative immobility time was scored for 6 min during the test. Sucrose preference test (SPT): Mice were habituated to a 1% sucrose solution for 24 h before the test day. Mice were deprived of water and food for 4 h from 13:00 pm to 17:00 pm, before a preference test for 1 h with water and 1% sucrose, which were provided in identical bottles. The bottles containing water and sucrose were weighed before and at the end of this period, and the sucrose preference (%) was determined.

### AAV viral production

A female Sprague Dawley rat (postnatal day 50, Japan, SLC, Hamamatsu, Japan) was deeply anaesthetized with isoflurane before being killed by cervical dislocation. EphA4 cDNA was cloned from the 1st strand cDNA generated from the frontal cortex of rat. The generated amplicon, which corresponded to nucleotides 57–3017 based on the numbering of NM_001162411, was fused to the *N*-terminus of monomeric Venus. PGK promoter::EphA4-Venus was packaged by the AAV helper-free system (Agilent Technologies, Santa Clara, CA), as described previously^64^. Briefly, pRep-Cap and the pHelper plasmid were co-transfected into AAV-293 cells. Cells were harvested and lysed with five freeze-thaw cycles. The resultant supernatants were subjected to CsCl gradient centrifugation. The virus-rich fraction was retained and the solvent was replaced with ASCF (1 mM MgCl_2_, 10 mM HEPES, CaCl_2_-free). The viral titer was determined by quantitative real-time PCR analysis (SYBR Green; Takara Bio Inc., Shiga, Japan).

### Virus injection

Male C57BL/6 mice (5 weeks old; n = 40) were used in the experiment. Subcutaneous injections of ketoprofen (40 µg per g of body weight) and penicillin/streptomycin (4 U per g body weight) were administered for four consecutive days from one day before the operation to prevent inflammation. On the day of surgery, the mice were anesthetized with pentobarbital (5 mg/ml; 0.1 ml/mouse), and mannitol (4 µg per g of body weight) and dexamethasone (7 µg per g of body weight) were administered intraperitoneally to prevent brain swelling, and the mice were placed in a stereotaxic frame. Microinjection needles were placed bilaterally into the mPFC (+1.7 AP, ±0.4 ML, −1.8 DV)^65^. Next, 1 μL of AAV (1.0 × 10^13^ genome copies/ml) was injected bilaterally. Behavioral evaluation was performed 3 weeks after the final infusion. Animals were deeply anaesthetized with isoflurane before being killed by cervical dislocation. Subsequently, brain regions were collected.

### Western blot

The brain samples of prefrontal cortex (PFC), CA1, CA3, dentate gyrus (DG) of the hippocampus, and nucleus accumbens (NAc) were prepared as described previously^30, 47, 50^. Western blot analyses were performed by two observers who were blinded to the group assignment of animals. Postmortem brain samples from control, depression, schizophrenia, and bipolar disorder subjects were obtained from the Neuropathology Consortium of the Stanley Medical Research Institute^28, 29, 66^. Western blot analysis was performed as described previously^30, 47, 50^. Basically, the tissue samples were homogenized in Laemmli lysis buffer. Aliquots (10 μg for mouse and 50 μg for human samples) of protein were measured using a DC protein assay kit (Bio-Rad, Hercules, CA) and incubated for 5 min at 95 °C with an equal volume of a mixture of 125 mM Tris/HCl, pH 6.8, 20% glycerol, 0.1% bromophenol blue, 10% β-mercaptoethanol, and 4% sodium dodecyl sulfate, then and subjected to sodium dodecyl sulfate-polyacrylamide gel electrophoresis, using 10% mini-gels (Mini-PROTEAN® TGX™ Precast Gel; Bio-Rad, CA, USA). Proteins were transferred onto polyvinylidene difluoride membranes using a Trans Blot Mini Cell (Bio-Rad). For immunodetection, the blots were blocked with 2% bovine serum albumin in TBST (Tris-buffered saline + 0.1% Tween-20) for 1 h at room temperature (RT), and incubated overnight at 4 °C with primary antibodies. The following primary antibodies were used: EphA4 (1:200, Santa Cruz Biotechnology, Inc., CA, USA), p-EphA4 (1:1000, ECM Bioscience), Ephexin1 (1:1000, Abcam, Cambridge, MA, USA), p-Ephexin1 (1:1000, Abcam), p-Cdk5 Tyr 15 (1:200, Santa Cruz Biotechnology, Inc.), Cdk5 (1:200, Santa Cruz Biotechnology, Inc.), BDNF (1:1000, Santa Cruz Biotechnology, Inc.), p-TrkB (Tyr 706) (1:200, Santa Cruz Biotechnology, Inc.), TrkB (80E3) (1:1000, Cell Signaling Technology, MA, USA), p-Fyn (Y530) (1:500, Abcam), Fyn (1:1000, Abcam), and PSD-95 (1 µg/ml, Invitrogen, Carlsbad, CA, USA). The next day, the blots were washed three times with TBST and incubated with horseradish peroxidase conjugated anti-rabbit antibody (1:1000 for EphA4, p-EphA4, Ephexin1, p-Ephexin1, Cdk5, and TrkB, and 1:5000 for BDNF), donkey anti-goat antibody (1:2000 for p-Cdk5), and goat anti-rabbit antibody (1:2000 for p-TrkB) for 1 hour at RT. After three final washes with TBST, the bands were detected using enhanced chemiluminescence plus the Western Blotting Detection system (GE Healthcare Bioscience). The blots then were washed three times with TBST and incubated with a primary antibody against β-actin. Images were captured with a Fuji LAS3000-mini imaging system (Fujifilm, Tokyo, Japan) and immunoreactive bands were quantified.

### Statistical analysis

The results were expressed as the mean ± standard error of the mean (S.E.M.). Analyses were performed using PASW Statistics 20 (formerly SPSS Statistics; SPSS). Comparisons between groups were performed using the Student’s *t*-test, one-way analysis of variance (ANOVA) followed by the *post-hoc* Tukey test, or two-way ANOVA, as appropriate. *Post hoc* comparisons were performed using the unpaired Student’s *t*-test. P-values less than 0.05 were considered to indicate significant differences.

## Electronic supplementary material

Supplemental information

## References

[1] World Health Organization (WHO). Depression Fact sheet No. 369/October 2012; Available at http://www.who.int/mediacentre/factsheets/fs369/en/index.html.

[2] Whooley MA, Simon GE (2000). Managing depression in medical outpatients. N Eng J Med.

[3] Rush AJ (2011). Combining medications to enhance depression outcomes (CO-MED): acute and long-term outcomes of a single-blind randomized study. Am J Psychiatry.

[4] Guidi J, Tomba E, Fava GA (2016). The sequential integration of pharmacotherapy and psychotherapy in the treatment of major depressive disorder: A meta-analysis of the sequential model and a critical review of the literature. Am J Psychiatry.

[5] Hashimoto K (2015). Inflammatory biomarkers as differential predictors of antidepressant response. Int J Mol Sci.

[6] Monteggia LM, Zarate C (2015). Antidepressant actions of ketamine: from molecular mechanisms to clinical practice. Curr Opin Neurobiol.

[7] Himanen JP, Nikolov DB (2003). Eph signaling: a structural view. Trends Neurosci.

[8] Martínez A, Soriano E (2005). Functions of ephrin/Eph interactions in the development of the nervous system: emphasis on the hippocampal system. Brain Res Brain Res Rev.

[9] Lai KO, Ip NY (2009). Synapse development and plasticity: roles of ephrin/Eph receptor signaling. Curr Opin Neurobiol.

[10] Tremblay ME (2007). Localization of EphA4 in axon terminals and dendritic spines of adult rat hippocampus. J Comp Neurol.

[11] Bouvier D (2008). Pre-synaptic and post-synaptic localization of EphA4 and EphB2 in adult mouse forebrain. J Neurochem.

[12] Pasquale EB (2008). Eph-ephrin bidirectional signaling in physiology and disease. Cell.

[13] Dalva MB (2000). EphB receptors interact with NMDA receptors and regulate excitatory synapse formation. Cell.

[14] Murai KK, Pasquale EB (2011). Eph receptors and ephrins in neuron-astrocyte communication at synapses. Glia.

[15] Hashimoto K (2009). Emerging role of glutamate in the pathophysiology of major depressive disorder. Brain Res Rev.

[16] Hashimoto K (2013). Sigma-1 receptor chaperone and brain-derived neurotrophic factor: emerging links between cardiovascular disease and depression. Prog Neurobiol.

[17] Gerhard DM, Wohleb ES, Duman RS (2016). Emerging treatment mechanisms of depression: focus on glutamate and synaptic plasticity. Drug Discov Today.

[18] Chen Y, Fu AKY, Ip NY (2012). Eph receptors at synapses: Implications in neurodegenerative diseases. Cell Signal.

[19] Fu WY (2006). Cdk5 regulates EphA4-mediated dendritic spine retraction through an ephexin1-dependent mechanism. Nat Neurosci.

[20] Fu AKY, Ip NY (2007). Cyclin-dependent kinase 5 links extracellular cues to actin cytoskeleton during dendritic spine development. Cell Adh Migr.

[21] Shi L, Fu AKY, Ip NY (2010). Multiple roles of the Rho GEF ephexin1 in synapse remodeling. Commun Integr Biol.

[22] McEwen BS (2007). Physiology and neurobiology of stress and adaptation: central role of the brain. Physiol Rev.

[23] Duman RS, Aghajanian GK (2012). Synaptic dysfunction in depression: Potential therapeutic targets. Science.

[24] Ohgi Y, Futamura T, Hashimoto K (2015). Glutamate signaling in synaptogenesis and NMDA receptors as potential therapeutic targets for psychiatric disorders. Curr Mol Med.

[25] Fu AK (2014). Blockade of EphA4 signaling ameliorates hippocampal synaptic dysfunctions in mouse models of Alzheimer’s disease. Proc Natl Acad Sci USA.

[26] Ng YP, Or TC, Ip NY (2015). Plant alkaloids as drug leads for Alzheimer’s disease. Neurochem Int.

[27] Hashimoto K (2015). Role of EphA4 in the pathogenesis of amyotrophic lateral sclerosis and therapeutic potential of traditional Chinese medicine rhynchophylline. Psychopharmacology (Berl).

[28] Ren Q (2016). Gene deciency and pharmacological inhibition of soluble epoxide hydrolase confers resilience to repeated social defeat stress. Proc Natl Acad Sci USA.

[29] Torrey EF, Webster M, Knable M, Johnston N, Yolken RH (2000). The stanley foundation brain collection and neuropathology consortium. Schizophr Res.

[30] Zhang JC (2016). Depression-like phenotype by deletion of α7 nicotinic acetylcholine receptor: Role of BDNF-TrkB in nucleus accumbens. Sci Rep.

[31] Nestler EJ (2002). Neurobiology of depression. Neuron.

[32] Hashimoto K, Shimizu E, Iyo M (2004). Critical role of brain-derived neurotrophic factor in mood disorders. Brain Res Brain Res Rev.

[33] Hashimoto K (2010). Brain-derived neurotrophic factor as a biomarker for mood disorders: an historical overview and future directions. Psychiatry Clin Neurosci.

[34] Hashimoto K, Malchow B, Falkai P, Schmitt A (2013). Glutamate modulators as potential therapeutic drugs in schizophrenia and affective disorders. Eur Arch Psychiatry Clin Neurosci.

[35] Castrén E (2014). Neurotrophins and psychiatric disorders. Handb Exp Pharmacol.

[36] Björkholm C, Monteggia LM (2015). BDNF- a key transducer of antidepressant effects. Neuropharmacology.

[37] Yang C, Shirayama Y, Zhang JC, Ren Q, Hashimoto K (2015). Regional differences in brain-derived neurotrophic factor levels and dendritic spine density confer resilience to inescapable stress. Int J Neuropsychopharmacol.

[38] Zhang JC (2015). Antidepressant effects of TrkB ligands on depression-like behavior and dendritic changes in mice after inflammation. Int J Neuropsychopharmacol.

[39] Li Y (2014). Differential expression of hippocampal EphA4 and ephrinA3 in anhedonic-like behavior, stress resilience, and antidepressant drug treatment after chronic unpredicted mild stress. Neurosci Lett.

[40] Marler KJ (2008). A TrkB/EphrinA interaction controls retinal axon branching and synaptogenesis. J Neurosci.

[41] Papadopoulou A (2015). Acute and chronic stress differentially regulate cyclin-dependent kinase 5 in mouse brain: implications to glucocorticoid actions and major depression. Transl Psychiatry.

[42] Zhu WL (2012). Increased Cdk5/p35 activity in the dentate gyrus mediates depressive-like behaviour in rats. Int J Neuropsychopharmacol.

[43] Zhong P (2014). Cyclin-dependent kinase 5 in the ventral tegmental area regulates depression-related behaviors. J Neurosci.

[44] Heller EA (2016). Targeted epigenetic remodeling of the Cdk5 gene in nucleus accumbens regulates cocaine- and stress-evoked behavior. J Neurosci.

[45] Dwivedi Y (2003). Altered gene expression of brain-derived neurotrophic factor and receptor tyrosin kinase B in postmortem brain of suicide subjects. Arch Gen Psychiatry.

[46] Karege F, Vaudan G, Schwald M, Perround N, La Harpe R (2005). Neurotrophin levels in postmortem brains of suicide victims and the effects of antemortem diagnosis and psychotropic drugs. Brain Res Mol Brain Res.

[47] Zhang JC (2015). Comparison of ketamine, 7,8-dihydroxyflavone, and ANA-12 antidepressant effects in the social defeat stress model of depression. Psychopharmacology.

[48] Shirayama Y (2015). Alterations in brain-derived neurotrophic factor (BDNF) and its precursor proBDNF in the brain regions of a learned helplessness rat model and the antidepressant effects of a TrkB agonist and antagonist. Eur Neuropsychopharmacol.

[49] Yang B (2016). Regional differences in the expression of brain-derived neurotrophic factor (BDNF) pro-peptide, proBDNF, and preproBDNF in the brain confer stress resilience. Eur Arch Psychiatry Clin Neurosci.

[50] Yang C (2015). R-ketamine: a rapid-onset and sustained antidepressant without psychotomimetic side effects. Transl Psychiatry.

[51] Krishnan V (2007). Molecular adaptations underlying susceptibility and resistance to social defeat in brain reward regions. Cell.

[52] Ren Q (2015). BDNF-TrkB signaling in the nucleus accunbens shell of mice has key role in methamphetamine withdrawal symptoms. Transl Psychiatry.

[53] Zhang JC, Yao W, Hashimoto K (2016). Brain-derived neurotrophic factor (BDNF)-TrkB signaling in inflammation-induced depression and potential therapeutic targets. Curr Neuropharmacol.

[54] Cheung ZH, Chin WH, Chen Y, Ng YP, Ip NY (2007). Cdk5 is involved in BDNF-stimulated dendritic growth in hippocampal neurons. PLoS Biol.

[55] Lai KO (2012). TrkB phosphorylation by Cdk5 is required for activity-dependent structural plasticity and spatial memory. Nat Neurosci.

[56] Forte A (2015). Long-term morbidity in bipolar-I, bipolar-II, and unipolar major depressive disorders. J Affect Disord.

[57] Sim K, Lau WK, Sim J, Sum MY, Baldessarini RJ (2015). Prevention of relapse and recurrence in adults with major depressive disorder: systematic review and meta-analyses of controlled trials. Int J Neuropsychopharmacol.

[58] Okamoto H (2014). A review of the evidence for use of this Kampo herbal formula in dementia and psychiatric conditions. Neuropsychitr Dis Treat.

[59] Kang TH (2002). Rhynchophylline and isorhynchophylline inhibit NMDA receptors expressed in Xenopus oocytes. Eur J Pharmacol.

[60] Kang TH (2004). Protective effect of rhynchophylline and isorhynchophylline on *in vitro* ischemia-induced neuronal damage in the hippocampus: putative neurotransmitter receptors involved in their action. Life Sci.

[61] Golden SA, Covington HE, Berton O, Russo SJ (2011). A standard protocol for repeated social defeat stress in mice. Nat Protoc.

[62] Zhao T (2013). Effects of chronic social defeat stress on behavior and choline acetyltransferase, 78-kDa glucose-regulated protein, and CCAAT/enhancer-binding protein (C/EBP) homologous protein in adult mice. Psychopharmacology.

[63] Walsh JJ (2014). Stress and CRF gate neural activation of BDNF in the mesolimbic reward pathway. Nature Neuroscience.

[64] Hayashi-Takagi A (2015). Labelling and optical erasure of synaptic memory traces in the motor cortex. Nature.

[65] Paxinos, G., Watson, C. The mouse brain in Stereotaxic Coordinates, The 4^th^ edition. Academic Press, San Diego, CA (1998).

[66] Yang B, Ren Q, Zhang JC, Chen QX, Hashimoto K (2017). Altered expression of BDNF, BDNF pro-peptide, and their precursor proBDNF in the brain and liver tissues from psychiatric disorders: rethinking the brain-liver axis. Transl Psychiatry.

