# Author Correction: Increased EphA4-ephexin1 signaling in the medial prefrontal cortex plays a role in depression-like phenotype

**DOI:** 10.1038/s41598-023-34971-6

**Published:** 2023-05-17

**Authors:** Ji-chun Zhang, Wei Yao, Youge Qu, Mayumi Nakamura, Chao Dong, Chun Yang, Qian Ren, Min Ma, Mei Han, Yukihiko Shirayama, Akiko Hayashi-Takagi, Kenji Hashimoto

**Affiliations:** 1 Division of Clinical Neuroscience, Chiba University Center for Forensic Mental Health, Chiba, 260-8670 Japan; 2 Laboratory of Medical Neuroscience, Institute for Molecular and Cellular Regulation, Gunma University, Gunma, 371-8511 Japan; 3 Department of Psychiatry, Teikyo University Chiba Medical Center, Ichihara, Chiba 299-0111 Japan; 4 PRESTO, Japan Science and Technology Agency, 4-1-8 Honcho, Kawaguchi, Saitama 332-0012 Japan

Correction to: *Scientific Reports* 10.1038/s41598-017-07325-2, published online 02 August 2017

This Article contains errors. The representative western blot bands do not agree with the original western blot data shown in the respective Supplementary Figures in the following original Figures:

- Figure 1b (DG), 1c (NAc and CA3), 1e (CA3 and DG);
- Figure 4c (PFC, NAc, and CA3), 4d (CA3), 4e (PFC, NAc, and DG), 4f (CA3);
- Figure 6g and 6k.

The corrected Figures 1, 4, and 6 and accompanying legends appear below.

**Figure 1:**
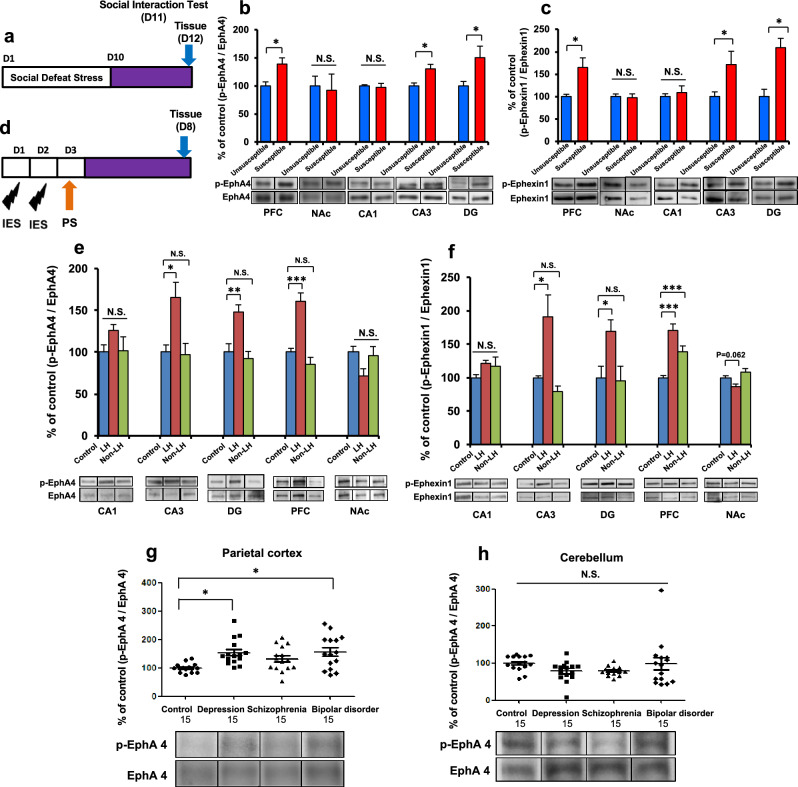
p-EphA4/EphA4 and p-ephexin1/ephexin1 ratios in the brain regions of mice with the depression-like phenotype and the postmortem brains of depressed patients. (**a**) Schedule of social defeat stress (10 days), social interaction test and brain sample collection. (**b** and **c**) The ratios of p-EphA4/EphA4 and p-ephexin1/ephexin1 in the mouse brain regions from social defeat stress (susceptible) mice and control (unsusceptible) mice. Data represent the mean ± S.E.M. (n = 5 or 6). *P < 0.05 compared with the control group. N.S.: not significant (Student’s *t*-test). (**d**) Schedule of learned helplessness (LH) and brain sample collection. (**e** and **f**) The ratios of p-EphA4/EphA4 and p-Ephexin1/Ephexin1 in the rat brain regions from control, LH, and non-LH rats (n = 5–6). Data represent the mean ± S.E.M. *P < 0.05, **P < 0.01 and ***P < 0.01 compared with the control group. N.S.: not significant. (**g** and **h**): The ratios of p-EphA4/EphA4 in the postmortem brain samples (parietal cortex and cerebellum) from control, depression, schizophrenia, and bipolar disorder subjects. Data represent the mean ± S.E.M. (n = 15). *P < 0.05 compared with control group, N.S.: not significant.

**Figure 4:**
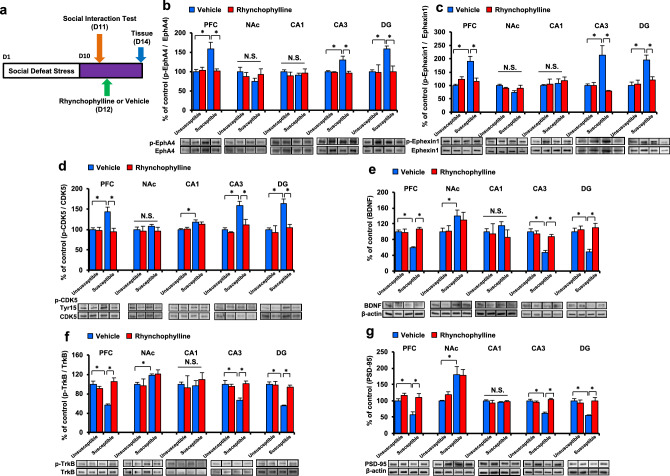
Role of EphA4-ephexin1-Cdk5 signaling, BDNF-TrkB signaling and synaptic protein in the antidepressant effect of rhynchophylline. (**a**) Schedule of social defeat stress (10 days), drug treatment, and brain sample collection. (**b**) Western blot analysis of EphA4 and p-EphA4 in the PFC, NAc, CA1, CA3, and DG of hippocampus. (**c**) Western blot analysis of ephexin1 and p-ephexin1 in the PFC, NAc, CA1, CA3, and DG of hippocampus. (**d**) Western blot analysis of p-Cdk5 (Tyr15)/Cdk5 in the PFC, NAc, CA1, CA3, and DG of hippocampus. The values are expressed as percentages relative to those in the control mice. The values represent the mean ± S.E.M. (n = 5 or 6). *P < 0.05 compared with the vehicle + stressed group. N.S.: not significant. (**e**): Western blot of BDNF in the PFC, NAc, CA1, CA3, and DG of hippocampus. (**f**): Western blot analysis of TrkB and p-TrkB in the PFC, NAc, CA1, CA3, and DG of hippocampus. (**g**): Western blot analysis of PSD-95 in the PFC, NAc, CA1, CA3, and DG of hippocampus. The values are expressed as percentages relative to those in the control mice. The values represent the mean ± S.E.M. (n = 5 or 6). *P < 0.05 compared with the vehicle + stressed group. N.S.: not significant.

**Figure 6:**
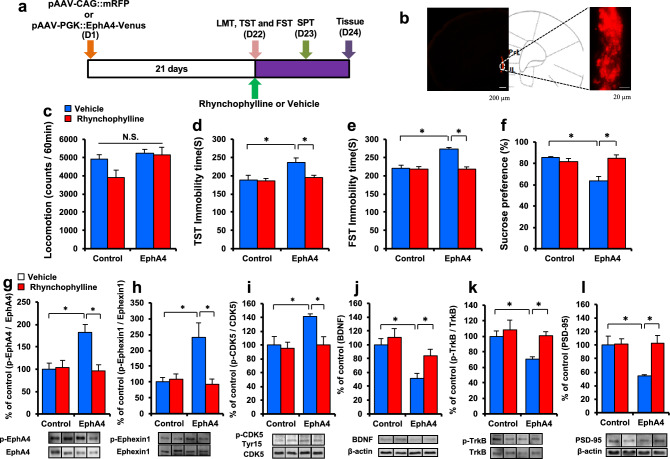
Depression-like phenotype after bilateral injection of pAAV-PGK:: EphA4-Venus into the mPFC. (**a**) Schedule of pAAV vector injection, drug treatment, and behavioral tests. (**b**) Representative photographs of the injection sites and coronal brain sections in the mPFC. The success of AAV vector injection into the mPFC was confirmed by the presence of mRFP fluorescence. Scale bars = 200 μm (low-power images) and 20 μm (high-power images). (**c**) LMT. (**d**) TST. (**e**) FST. (**f**) SPT. The values represent the mean ± S.E.M. (n = 7). *P < 0.05 compared with the vehicle + EphA4 group. N.S.: not significant. (**g**) p-EphA4/EphA4 ratio in the PFC. (**h**) p-Ephexin1/Ephexin1 ratio in the PFC. (**i**) p-Cdk5(Tyr15)/Cdk5 ratio in the PFC. (**j**) BDNF in the PFC. (**k**) p-TrkB/TrkB ratio in the PFC. (**l**) PSD-95 in the PFC. The values represent the mean ± S.E.M. (n = 5 or 6). *P < 0.05 compared with the vehicle + EphA4 group. N.S.: not significant.

In addition, the Supplementary Information file published with this Article contains errors.

- In Supplementary Figure S5 the same original blot is shown for P-EphA4 and P-TrkB;
- Supplementary Figure S4 is wrongly labelled to show the original western blots from Figure 3;
- Supplementary Figure S5 is wrongly labelled to show the original western data blots from Figure 5.

The corrected Supplementary Information file is provided below and is updated to include the following changes:

- The correct original western blot data for the P-EphA4 panel in Supplementary Figure S5A are provided;
- Supplementary Figure S4 indicates now that it contains the original western blot data from Figure 4;
- Supplementary Figure S5 indicates now that it contains the original western blot data from Figure 6;
- The original western blot data for Figures S1 and S2 are shown in Supplementary Figure S6.

## Supplementary Information

Supplementary Information.

