# Supplementary material for: Increased EphA4-ephexin1 signaling in the medial prefrontal cortex plays a role in depression-like phenotype
Source: Sci Rep. 2017 Aug 2;7:7133. doi: 10.1038/s41598-017-07325-2 (PMC5541046; doi:10.1038/s41598-017-07325-2)

Supplemental Information

Increased EphA4-ephexin1 signaling in the medial prefrontal cortex plays a role in depression-like phenotype

Ji-chun Zhang,[a] Wei Yao,[a] Youge Qu,[a] Mayumi Nakamura[b], Chao Dong,[a] Chun Yang,[a] Qian Ren,[a] Min Ma,[a] Mei Han,[a] Yukihiko Shirayama,[a,c] Akiko Hayashi-Takagi,[b,d] and Kenji Hashimoto[a,*]

Affiliations: [a]Division of Clinical Neuroscience, Chiba University Center for Forensic Mental Health, Chiba 260-8670, Japan; [b]Laboratory of Medical Neuroscience, Institute for Molecular and Cellular Regulation, Gunma University, Gunma 371-8511, Japan; [c]Department of Psychiatry, Teikyo University Chiba Medical Center, Ichihara, Chiba 299-0111, Japan; [d]PRESTO, Japan Science and Technology Agency, 4-1-8 Honcho, Kawaguchi, Saitama 332-0012, Japan

[*]**Corresponding author:** Dr. Kenji Hashimoto, Division of Clinical Neuroscience, Chiba University Center for Forensic Mental Health, 1-8-1 Inohana, Chiba 260-8670, Japan. Tel: +81-43-226-2587 (hashimoto@faculty.chiba-u.jp)

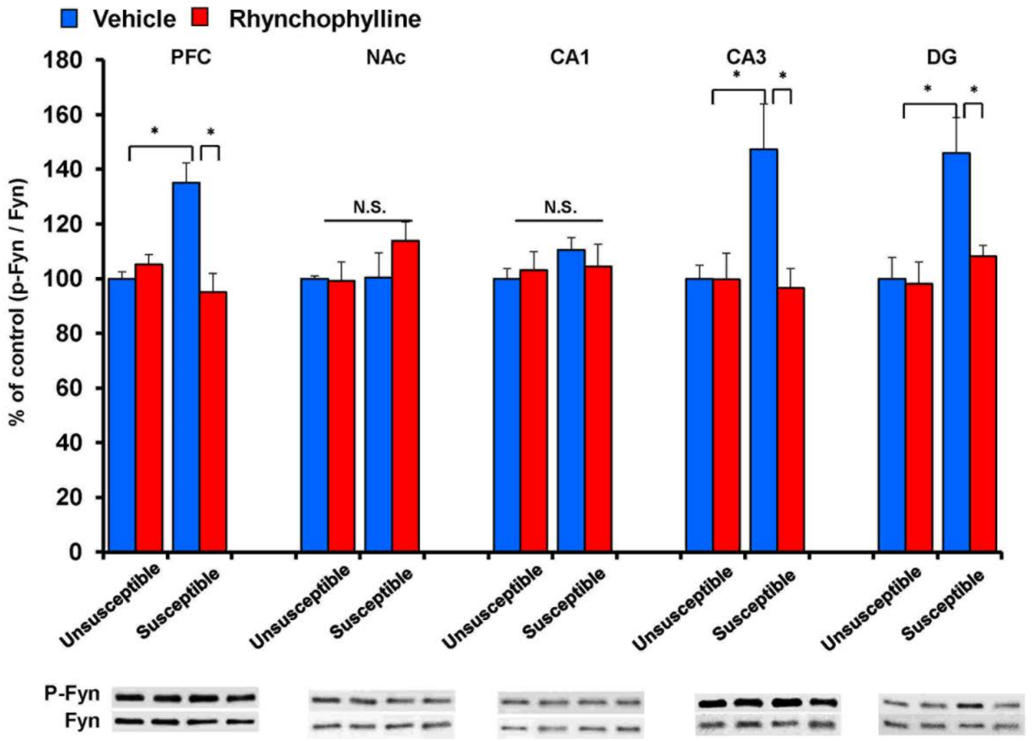

Figure S1. The p-Fyn/Fyn ratio in the brain regions of susceptible mice after social defeat stress. The values represent the mean ± S.E.M. (n = 6). *P < 0.05 compared with the vehicle + susceptible group. N.S.: not significant.

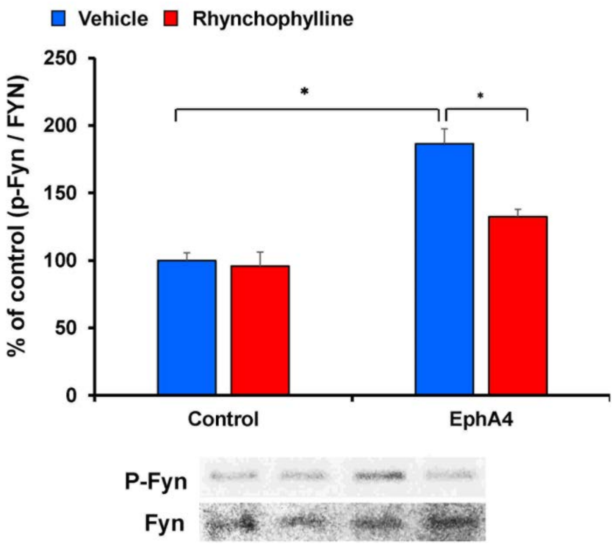

Figure S2. The p-Fyn/Fyn ratio in the PFC of mice after AAV vector injection. The values represent the mean ± S.E.M. (n = 6). *P < 0.05 compared with the vehicle + EphA4 group.

Figure S3. The original blots of Western blot analyses in the figure 1.

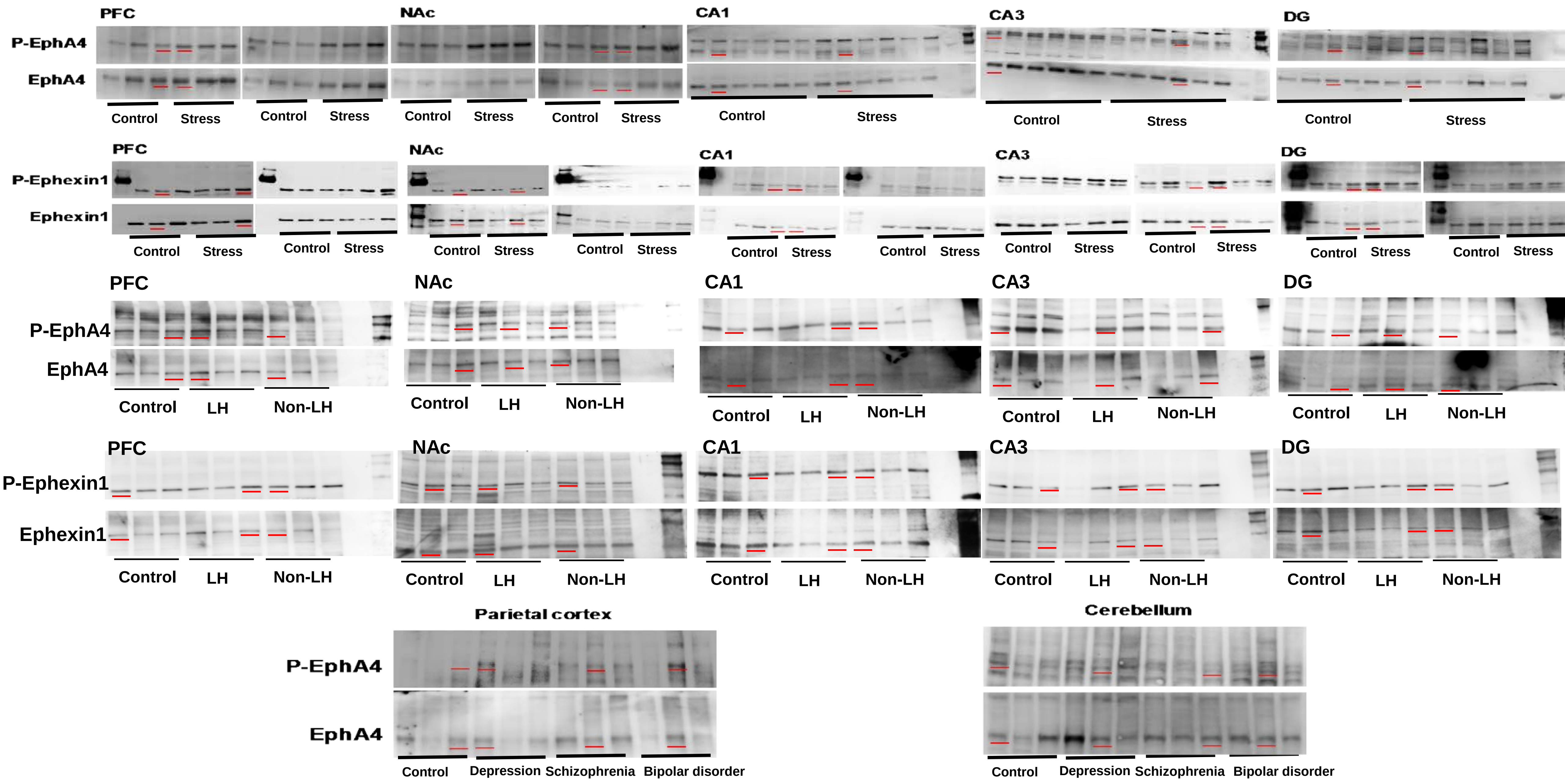

Figure S4. The original blots of Western blot analyses in the figure 3.

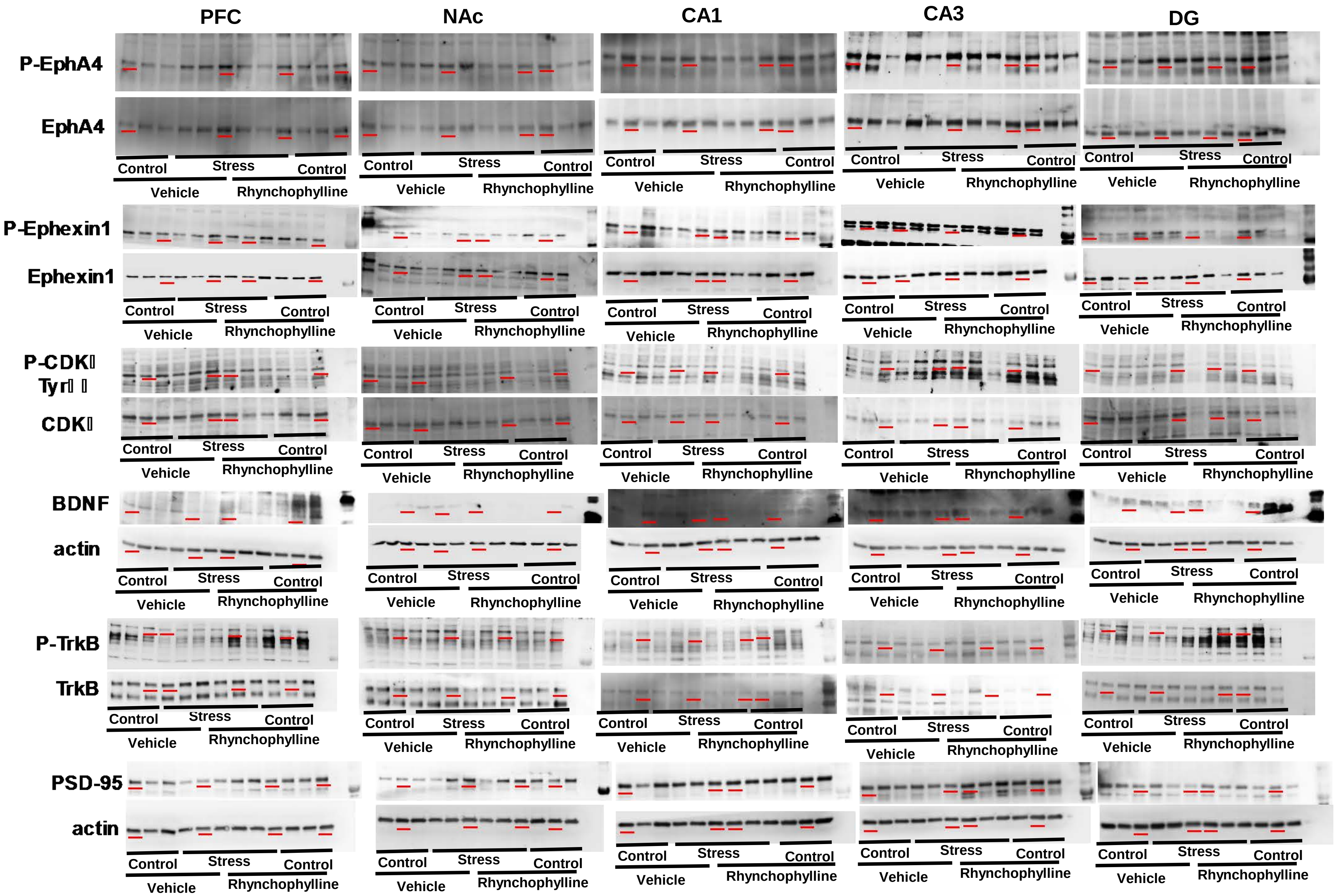

Figure S5. The original blots of Western blot analyses in the figure 5.

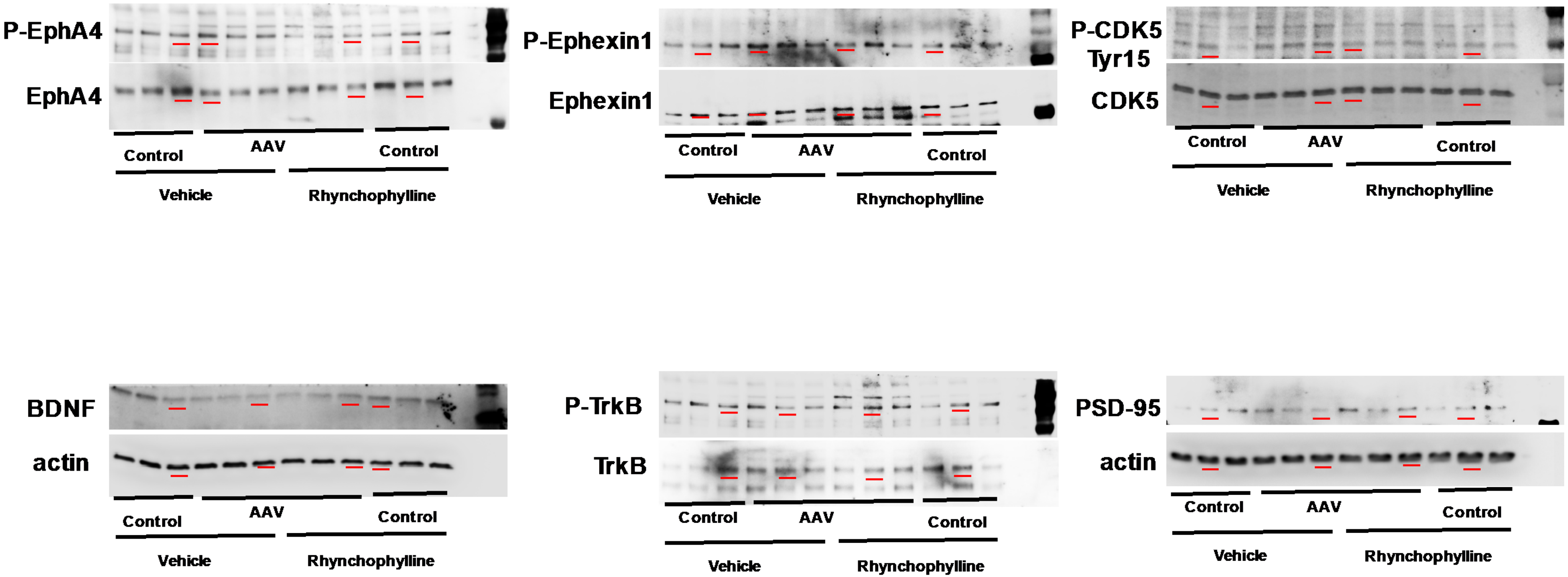

Supplement: Supplementary file 1 — Supplemental information [file 41598_2017_7325_MOESM1_ESM.pdf]
