# Supplementary material for: Author Correction: Increased EphA4-ephexin1 signaling in the medial prefrontal cortex plays a role in depression-like phenotype
Source: Sci Rep. 2023 May 17;13:8059. doi: 10.1038/s41598-023-34971-6 (PMC10192427; doi:10.1038/s41598-023-34971-6)
Supplement: Supplementary file 1 — Supplementary Information. [file 41598_2023_34971_MOESM1_ESM.pdf]

Supplemental Information

Increased EphA4-ephexin1 signaling in the medial prefrontal cortex plays a role in depression-like phenotype

Ji-chun Zhang,[a] Wei Yao,[a] Youge Qu,[a] Mayumi Nakamura[b], Chao Dong,[a] Chun Yang,[a] Qian Ren,[a] Min Ma,[a] Mei Han,[a] Yukihiko Shirayama,[a,c] Akiko Hayashi-Takagi,[b,d] and Kenji Hashimoto[a,*]

Affiliations: [a]Division of Clinical Neuroscience, Chiba University Center for Forensic Mental Health, Chiba 260-8670, Japan; [b]Laboratory of Medical Neuroscience, Institute for Molecular and Cellular Regulation, Gunma University, Gunma 371-8511, Japan; [c]Department of Psychiatry, Teikyo University Chiba Medical Center, Ichihara, Chiba 299-0111, Japan; [d]PRESTO, Japan Science and Technology Agency, 4-1-8 Honcho, Kawaguchi, Saitama 332-0012, Japan

[*]**Corresponding author:** Dr. Kenji Hashimoto, Division of Clinical Neuroscience, Chiba University Center for Forensic Mental Health, 1-8-1 Inohana, Chiba 260-8670, Japan. Tel: +81-43-226-2587 (hashimoto@faculty.chiba-u.jp)

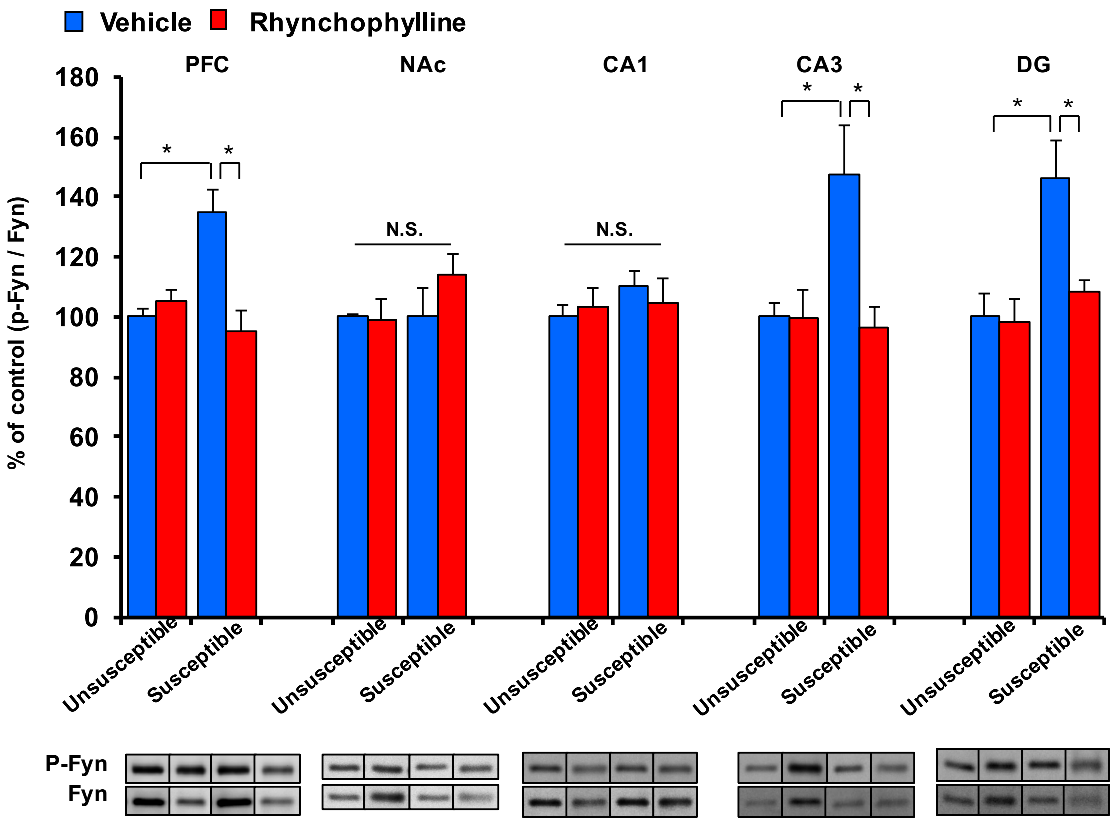

Figure S1. The p-Fyn/Fyn ratio in the brain regions of susceptible mice after social defeat stress. The values represent the mean ± S.E.M. (n = 6). *P < 0.05 compared with the vehicle + susceptible group. N.S.: not significant.

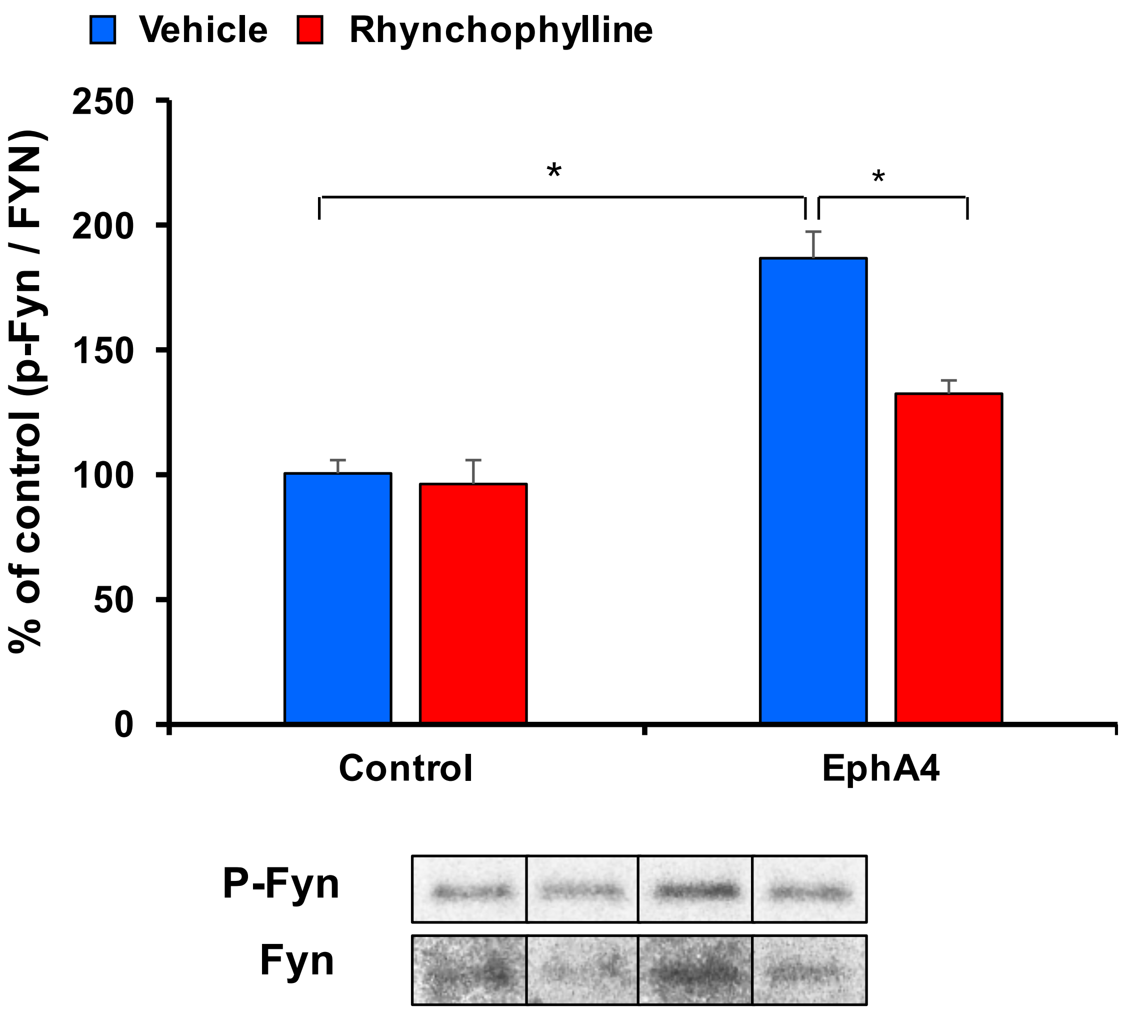

Figure S2. The p-Fyn/Fyn ratio in the PFC of mice after AAV vector injection. The values represent the mean ± S.E.M. (n = 6). *$P < 0.05$ compared with the vehicle + EphA4 group.

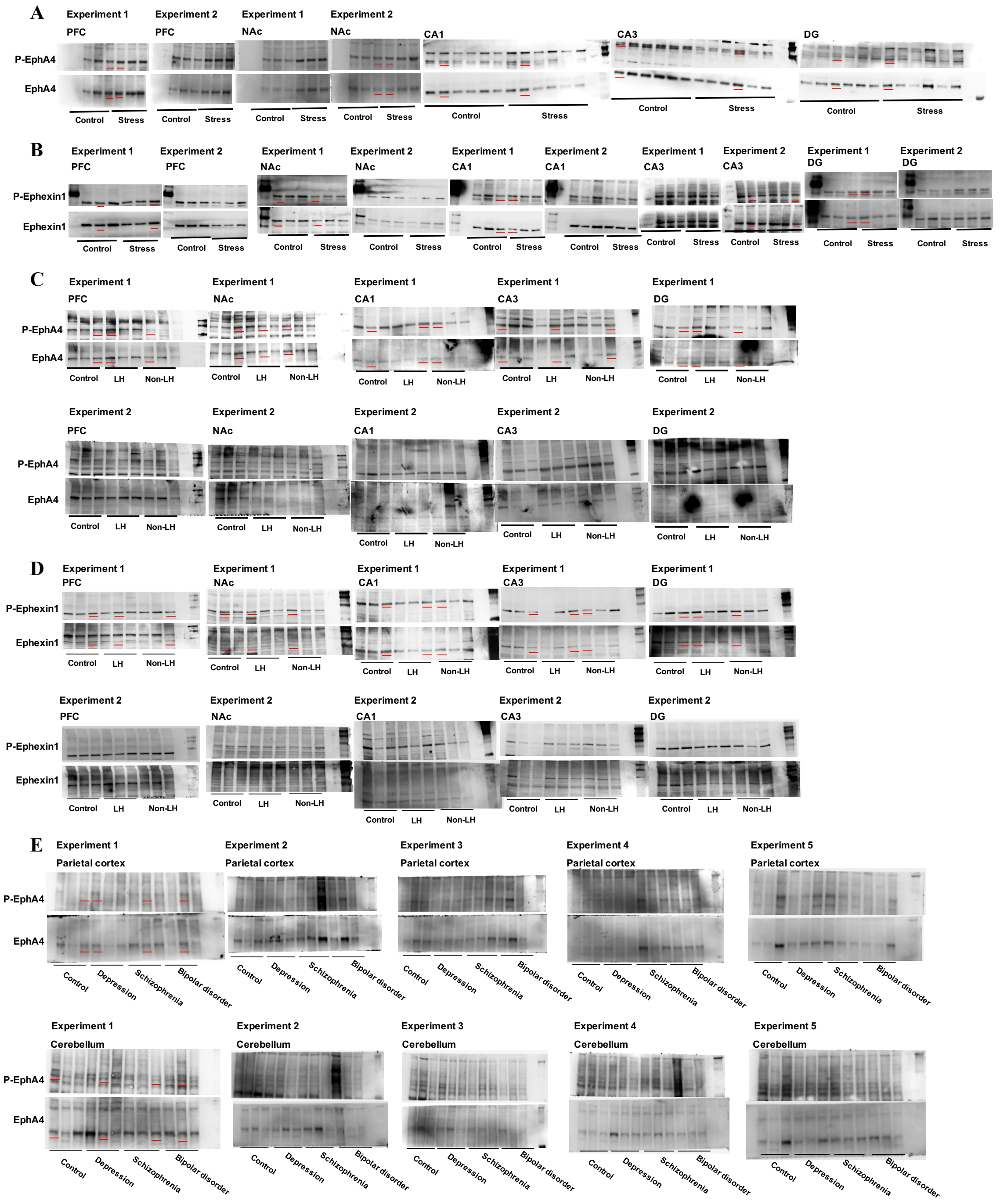

Figure S3. The original blots of Western blot analyses in the figure 1. The bands with red line were used as the representative bands of figure 1. A is the raw data for figure 1b. B is the raw data for figure 1c. C is the raw data for figure 1e. D is the raw data for figure 1f. E is the raw data for figure 1g and h.

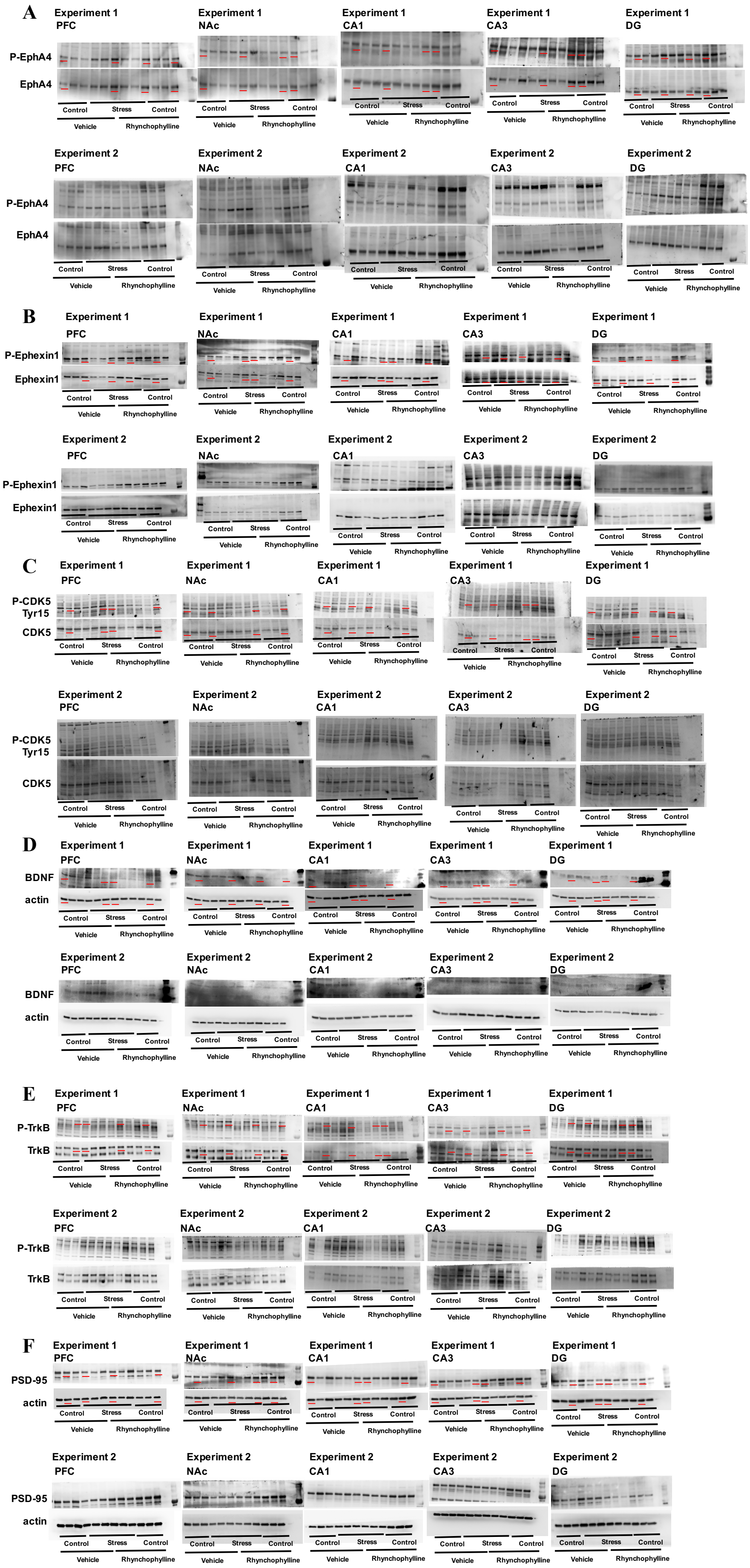

A
Experiment 1 PFC
Experiment 1 NAc
Experiment 1 CA1
Experiment 1 CA3
Experiment 1 DG
P-EphA4
EphA4
Control Stress Control
Vehicle Rhynchophylline
Experiment 2 PFC
Experiment 2 NAc
Experiment 2 CA1
Experiment 2 CA3
Experiment 2 DG
P-EphA4
EphA4
Control Stress Control
Vehicle Rhynchophylline
B
Experiment 1 PFC
Experiment 1 NAc
Experiment 1 CA1
Experiment 1 CA3
Experiment 1 DG
P-Ephexin1
Ephexin1
Control Stress Control
Vehicle Rhynchophylline
Experiment 2 PFC
Experiment 2 NAc
Experiment 2 CA1
Experiment 2 CA3
Experiment 2 DG
P-Ephexin1
Ephexin1
Control Stress Control
Vehicle Rhynchophylline
C
Experiment 1 PFC
Experiment 1 NAc
Experiment 1 CA1
Experiment 1 CA3
Experiment 1 DG
P-CDK5 Tyr15
CDK5
Control Stress Control
Vehicle Rhynchophylline
Experiment 2 PFC
Experiment 2 NAc
Experiment 2 CA1
Experiment 2 CA3
Experiment 2 DG
P-CDK5 Tyr15
CDK5
Control Stress Control
Vehicle Rhynchophylline
D
Experiment 1 PFC
Experiment 1 NAc
Experiment 1 CA1
Experiment 1 CA3
Experiment 1 DG
BDNF
actin
Control Stress Control
Vehicle Rhynchophylline
Experiment 2 PFC
Experiment 2 NAc
Experiment 2 CA1
Experiment 2 CA3
Experiment 2 DG
BDNF
actin
Control Stress Control
Vehicle Rhynchophylline
E
Experiment 1 PFC
Experiment 1 NAc
Experiment 1 CA1
Experiment 1 CA3
Experiment 1 DG
P-TrkB
TrkB
Control Stress Control
Vehicle Rhynchophylline
Experiment 2 PFC
Experiment 2 NAc
Experiment 2 CA1
Experiment 2 CA3
Experiment 2 DG
P-TrkB
TrkB
Control Stress Control
Vehicle Rhynchophylline
F
Experiment 1 PFC
Experiment 1 NAc
Experiment 1 CA1
Experiment 1 CA3
Experiment 1 DG
PSD-95
actin
Control Stress Control
Vehicle Rhynchophylline
Experiment 2 PFC
Experiment 2 NAc
Experiment 2 CA1
Experiment 2 CA3
Experiment 2 DG
PSD-95
actin
Control Stress Control
Vehicle Rhynchophylline

Figure S4. The original blots of Western blot analyses in the figure 4. The bands with red line were used as the representative bands of figure 4. A is the raw data for figure 4b. B is the raw data for figure 4c. C is the raw data for figure 4d. D is the raw data for figure 4e. E is the raw data for figure 4f. F is the raw data for figure 4g.

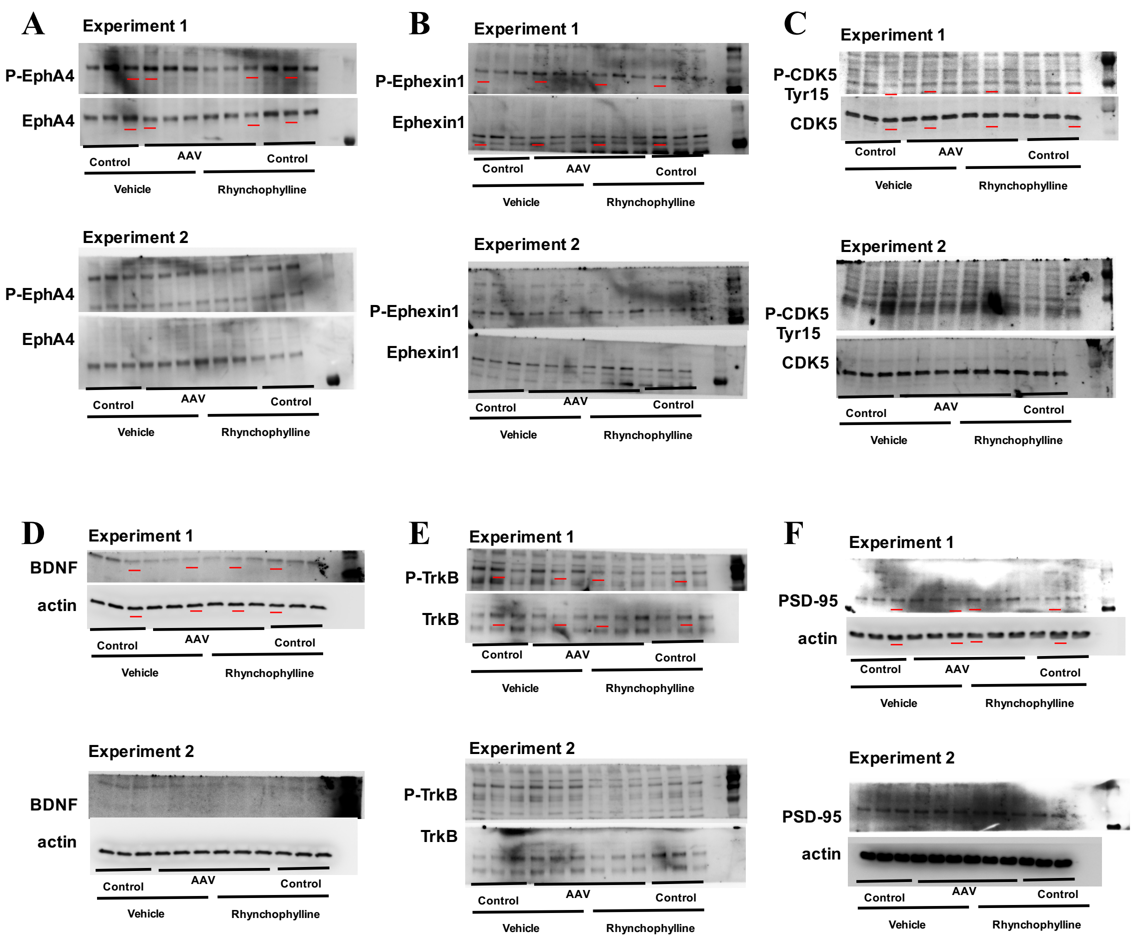

Figure S5. The original blots of Western blot analyses in the figure 6. The bands with red line were used as the representative bands of figure 6. A is the raw data for figure 6g. B is the raw data for figure 6h. C is the raw data for figure 6i. D is the raw data for figure 6j. E is the raw data for figure 6k. F is the raw data for figure 6l.

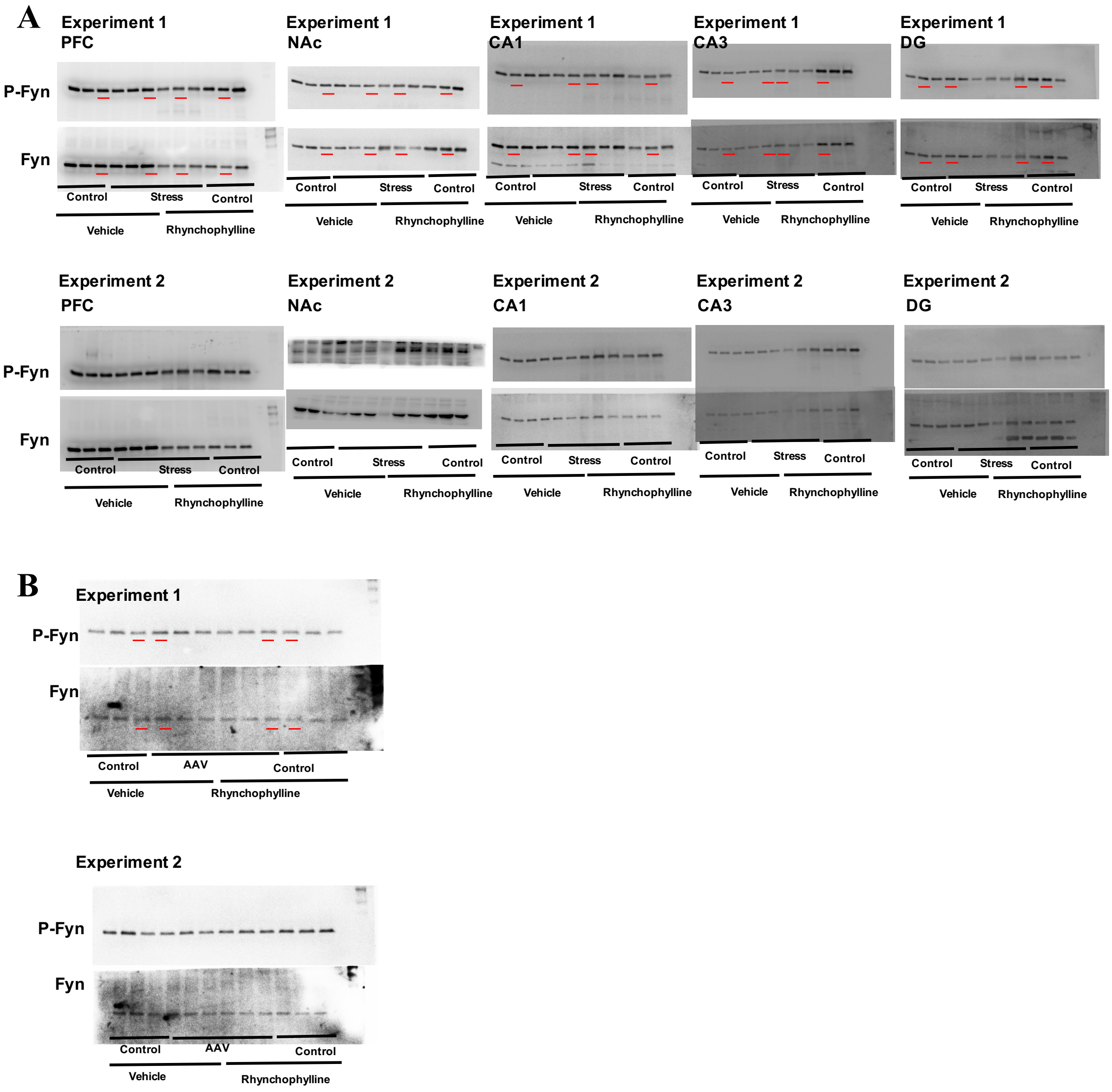

Figure S6. The original blots of Western blot analyses in the figure S1 and figure S2. The bands with red line were used as the representative bands of figure S1 and figure S2. A is the raw data for figure S1. B is the raw data for figure S2.
